# The Immune Environment in Colorectal Adenoma: A Systematic Review

**DOI:** 10.3390/biomedicines13030699

**Published:** 2025-03-12

**Authors:** Ugne Silinskaite, Jurate Valciukiene, Matas Jakubauskas, Tomas Poskus

**Affiliations:** Institute of Translational Health Research, Faculty of Medicine, Vilnius University, LT-03101 Vilnius, Lithuania

**Keywords:** colorectal, adenoma, carcinoma, adenoma–carcinoma sequence, immune infiltration, immune cell, cytokine, carcinogenesis

## Abstract

**Background/Objectives:** Research on colorectal adenoma is significantly less comprehensive compared to studies on colorectal carcinoma. Although colorectal adenoma is a precursor of the majority of sporadic colorectal cancers, not all adenomas develop into carcinomas. The complex interaction of immune responses in the premalignant tumor microenvironment might be a factor for that. **Methods:** In this systematic review, we aim to provide a thorough analysis of the current research examining the immune infiltration patterns in sporadic colorectal adenoma tissues in the context of immune cell-based, cytokine-based, and other immunological factor-related changes along the conventional adenoma–carcinoma sequence. The articles included in the review extend up to December 2024 in PubMed and Web of Science databases. **Results:** Most included studies have shown significant differences in immune cell counts, densities, and cytokine expression levels associated with premalignant colorectal lesions (and/or colorectal cancer). No consensus on the immune-related tendencies concerning CD4+T cells and CD8+T cells was reached. Decreasing expression of mDCs and plasma and naïve B cells were detected along the ACS. The increased density of tissue eosinophils in the adenoma tissue dramatically diminishes after the transition to carcinoma. As the adenoma progresses, the increasing expression of IL-1α, IL-4, IL-6, IL-8, IL-10, IL-17A, IL-21, IL-23, IL-33, and TGF-β and decreasing levels of IL-12A, IL-18, IFN—γ, and TNFα cytokines in the invasive carcinoma stage is being detected. The over-expression of COX-2, PD-1/PD-L1, CTLA-4, and ICOS/ICOSLG in the colorectal adenomatous and cancerous tissues was also observed. **Conclusions:** Further studies are needed for a better understanding of the whole picture of colorectal adenoma-associated immunity and its impact on precancerous lesion’s potential to progress.

## 1. Introduction

With more than 1.9 million new cases, colorectal cancer (CRC) ranks third among all cancers, as estimated in 2022. Moreover, it is the second most common cause of cancer-related death for men and women worldwide, accounting for approximately 904,000 deaths annually [1]. It is widely accepted that the main precursor of CRC is a dysplastic polypoid lesion of colorectum–sporadic colorectal (CR) adenoma [2,3,4]. In the conventional adenoma–carcinoma sequence (ACS), a stepwise transition occurs that is characterized by the accumulation of genetic and epigenetic mutations, changes in gut microbiota composition, and shifts in local immunity profile [4,5]. The conventional chromosomal instability (CIN) pathway typically begins with a mutation in the adenomatous polyposis coli (APC) gene, followed by mutations in KRAS, PIK3CA, and SMAD4 as well as the loss of heterozygosity due to p53 mutation [3,5]. Several risk factors have also been associated with the multistep complex process of CR tumorigenesis. These include a diet high in processed meats, low in fruits and vegetables, a sedentary lifestyle, obesity, smoking, excessive alcohol consumption, age (particularly individuals over 50), chronic inflammation in the colorectum, and hereditary colon syndromes, such as familial adenomatous polyposis (FAP) or Lynch syndrome (hereditary nonpolyposis colorectal cancer (HNPCC)) and others [5,6,7,8].

The well-established paradigm suggests that colorectal carcinoma typically develops from colorectal adenomas, which can exhibit low- or high-grade dysplasia [2]. This progression occurs in multiple steps, starting from healthy epithelial tissue, which transforms into aberrant crypt foci (ACF) and then into early and advanced adenomatous polyps. These polyps can advance to intramucosal carcinoma, followed by early colorectal cancer, eventually leading to invasive adenocarcinoma [2,3,4,9]. However, it is important to note that only a small percentage of early colorectal adenomas will ultimately progress to invasive colorectal cancer [9,10]. Recent research indicates that the complex balance of immune function within the tumor immune microenvironment (TiME) may play a significant role in this phenomenon [11,12].

The TiME is defined as a specific lesion-related milieu where tumor cells have multiple and consistent interactions with host immune cells, endothelial cells, fibroblasts, extracellular matrix, etc. [13,14]. In this setting, the local immune system plays a dual role. On one hand, certain immune cells produce cytokines that help maintain immunosurveillance and inhibit the progression of premalignant lesions. On the other hand, another type of immune cell may switch the immune response to an immunosuppressive mode, directly promoting tumor growth and progression [14,15,16].

Initially, the TiME was extensively studied in malignant colorectal lesions, revealing significant differences in immune responses between CRC tissues and healthy controls [17,18]. Numerous studies have shown that tumor-infiltrating lymphocytes (TILs) play a significant role in the prognosis [18] of colorectal carcinoma and the effectiveness of immunotherapy [19,20]. The type and distribution of tumor-infiltrating lymphocytes (TILs) are important prognostic factors for colorectal cancer. Some researchers propose that indicators of tumor immunity are more closely related to CRC survival than the clinical stage of the disease [21]. For instance, higher levels of T helper (Th) 1 cells, cytotoxic CD8+ T cells, and natural killer (NK) cells are associated with lower recurrence rates and better outcomes [22,23,24]. Conversely, greater infiltration of inflammatory T helper (Th) 17 cells or increased levels of interleukin-17A (IL-17A) are linked to a poorer prognosis [25].

The increased immunogenic response is often linked to elevated cytokine levels. Proinflammatory cytokines like interleukin-1 (IL-1), tumor necrosis factor-alpha (TNF-α), and interleukin-6 (IL-6) promote cell survival [26,27], while transforming growth factor-beta (TGF-β) suppresses immune responses and reduces cell apoptosis. Reactive oxygen species from myeloid-derived cells may induce mutations in tumor cells [28]. Some researchers introduced cytokines disseminated throughout the circulatory system as potential biomarkers for the diagnosis and prognosis of CRC. In addition, immune-related cytokines could be useful in monitoring following therapy [29]. Conversely, others argue that circulating inflammatory cytokines lack the specificity and sensitivity necessary for an early diagnosis and accurate prognosis of cancerous CR lesions [30].

Colorectal adenoma, overall, is less studied regarding immune cells, cytokines, and other TiME components than invasive carcinoma [31]. Several studies have investigated the immune cell landscape in large CR adenomas [32,33,34,35]. The main findings indicated an increase in Th17-related cytokines and a decrease in Th1-associated cytokines, along with the infiltration of cytotoxic (CD8) T cells and natural killer (NK) cells throughout the ACS [32,35]. Research on mast cells [36,37] and NK cells [38] has highlighted their roles in the immune surveillance mechanisms during the early stages of CR carcinogenesis. However, conflicting results have been published regarding the pro- and antitumorigenic activities of these cells [37,38,39,40]. Colorectal adenomas, similar to CRC, have been found to contain and/or express interleukin 10 (IL-10)-producing regulatory T cells, along with an over-expression of CXCL8 (neutrophil-attracting inflammatory chemokine) [41]. The study on the neutrophils/lymphocyte ratio (NLR) revealed that patients with polyps larger than 10 mm tend to have significantly higher NLRs than those with up to 10 mm-sized polyps [42]. Moreover, several immune markers, chemokines, and related gene expressions have also been studied in the immune microenvironment of CR adenomatous polyps [43,44,45,46]. However, the results of such studies lack the united examination and evaluation methods, are restricted to small sample sizes, and avoid control group assessment; therefore, they should be interpreted with great care. Furthermore, research on tissue-associated eosinophilia and components of extracellular matrix in the colorectal adenoma-associated immune microenvironment is still scarce. In addition, there is a lack of data characterizing precancerous CR lesion-related immune infiltrates according to the histological type of the lesion, grade of dysplasia, and polyp location in the colorectum.

Considering all the aforementioned inconclusive analyses and, to some extent, discordant results on the immune landscape of premalignant CR lesions, there is an urgent need for a profound assessment of immune responses in sporadic CR adenoma and every step of the conventional adenoma–carcinoma (A–C) pathway. A deeper understanding of immune patterns in precancerous tumors may provide insights for immune prevention research, improve early diagnostic and prognostic strategies, and enhance the identification of novel immunotherapeutic approaches.

Here, we present the first attempt to systematically analyze the compositional and functional changes of immune infiltrates in the human microenvironment of sporadic precancerous colorectal lesions compared to invasive CRC tissue and adjacent normal mucosa and/or healthy controls. First, the count, density, gene expression, and/or distribution of tumor-infiltrating immune cells in the epithelial and stromal compartments as well as the cytokine expression level and/or their tendencies for an increase or decrease along the conventional ACS were analyzed among the studies included in the systematic review. Second, we aimed to determine how the sporadic colorectal neoplasia-related immune infiltration correlates with the lesion’s morphology, size, dysplasia grade, and location in the gut.

## 2. Materials and Methods

The present systematic review was performed according to the Cochrane collaboration-specific protocol [47] and was reported following the Preferred Reporting Items for Systematic Reviews and Meta-analyses (PRISMA) guidelines for 2020 [48]. The PRISMA checklist has been completed aligned with the recommendations (Table S1). The present systematic review was prospectively registered on the INPLASY register (registration no.: INPLASY202470121).

### 2.1. Eligibility Criteria

This review included human studies investigating immune cell-associated and/or cytokine-based and/or other immunological factor-related changes of local immune response in sporadic CR adenoma and along the stages of the A–C pathway.

The search was restricted to human studies, excluding research involving animals and organoids. The selected studies had to be published in English up to December 2024 and have full-text availability.

The studies included adult patients (≥18 years) with a pathologically confirmed sporadic CR adenomatous lesion (-as): low- or high-grade; early or advanced; tubular, tubulovillous, or villous; and protruded and flat adenoma, which varied in size and location in the colorectum. The analysis focused on studies with healthy subjects’ or the same patient’s normal mucosa samples as controls. Additionally, although lacking a control group, several studies were still included due to extraordinary relevance and novelty in the field. Besides colorectal adenoma, all included studies also examined sporadic adenocarcinoma, warranting the evaluation of immune pattern changes throughout every step of conventional ACS.

### 2.2. Information Sources

The PubMed and Web of Science (WOS) online databases were used for the literature search concerning the immune environment in conventional colorectal adenoma. The last search was conducted in December 2024. Eight external articles were identified from other sources.

### 2.3. Search Strategy

The advanced search function was used in the literature search for this systematic review. The search was carried out following the PICOS model (Table S2) and using Medical Subject Headings (MeSH) and keywords with the employment of “AND” or “OR” Boolean operators: “Colorectal adenoma” OR “Colorectal polyp” OR “Colorectal polypoid lesion” OR “Colorectal precancerous lesion” OR “Colorectal neoplasms” OR “Colorectal neoplasia” OR “Colonic neoplasia” OR “Colorectal premalignant lesion” OR “Dysplastic colorectal lesion” AND/OR “Colorectal carcinogenesis” OR “Colorectal tumorigenesis” OR “Adenoma-carcinoma sequence” OR “conventional pathway” OR “Adenoma-carcinoma pathway” AND “Immune infiltration” OR “Immune infiltrates” OR “Immune landscape” OR “Immune environment” OR “Immune response” OR “Immune profile” OR “Immune expression” OR “Immunity” OR “Immune cell” OR “Cytokine” OR “Tumor-infiltrating lymphocytes”.

### 2.4. Selection Process

After obtaining search results, two independent reviewers read the titles and abstracts of the studies provided independently of one another. If more clarity was required, the entire article was thoroughly examined. In case of a disagreement among the initial reviewers, the third reviewer–consultant was asked to look at the debatable full article text. Following the identification of relevant abstracts, full-text articles were retrieved and re-reviewed. Comments on articles, short notes, letters, conference abstracts, systematic reviews, meta-analyses, review articles, preclinical studies, and duplicates or triple records were manually excluded. A manual search was performed to identify additional primary studies and minimize search bias. The literature review was completed with an extensive search using PubMed’s “related articles” function.

Studies examining the immune infiltration in colitis-associated, serrated, or hereditary syndrome-related CR lesions were not selected for the systematic review. The endpoint measured the local immunity shifts in sporadic CRA tissues along the conventional CR carcinogenesis. Secondary endpoints included the correlation between immune changes and lesion-associated characteristics (morphology, grade of dysplasia, size, and location in the gut).

### 2.5. Data Collection Process

We extracted various data points, including authors’ names, years of publication, methodological details, sample sizes of participants/subjects (exposure and control groups), and main findings, which were organized into a Microsoft Excel spreadsheet for a comprehensive analysis (Table S3). To reduce selection bias, the extracted data were evaluated at the end of the review process.

### 2.6. Protocol Registration

The review protocol entitled “IMMUNE ENVIRONMENT IN COLORECTAL ADENOMAS: a systematic review” was registered on the INPLASY register. The registration number is INPLASY202470121. DOI number is 10.37766/inplasy2024.7.0121.

### 2.7. Study Quality Assessment and Risk of Bias

The methodological quality of the selected trials was assessed using the Cochrane Handbook method [47]. For evaluating the quality of non-randomized trials, the Newcastle–Ottawa scale (NOS) was employed [49]. We rated the quality of the studies by awarding stars in each domain as follows: a maximum of one star was given for each numbered item within the selection and exposure categories, and a maximum of two stars was given for comparability. Only good- and fair-quality studies (≥5/9) were included in the further analysis. A summary of the quality evaluation process has been visualized in Table 1 and Table S4 (extended version).

## 3. Results

### Search Results and Study Characteristics

**Figure 1:**
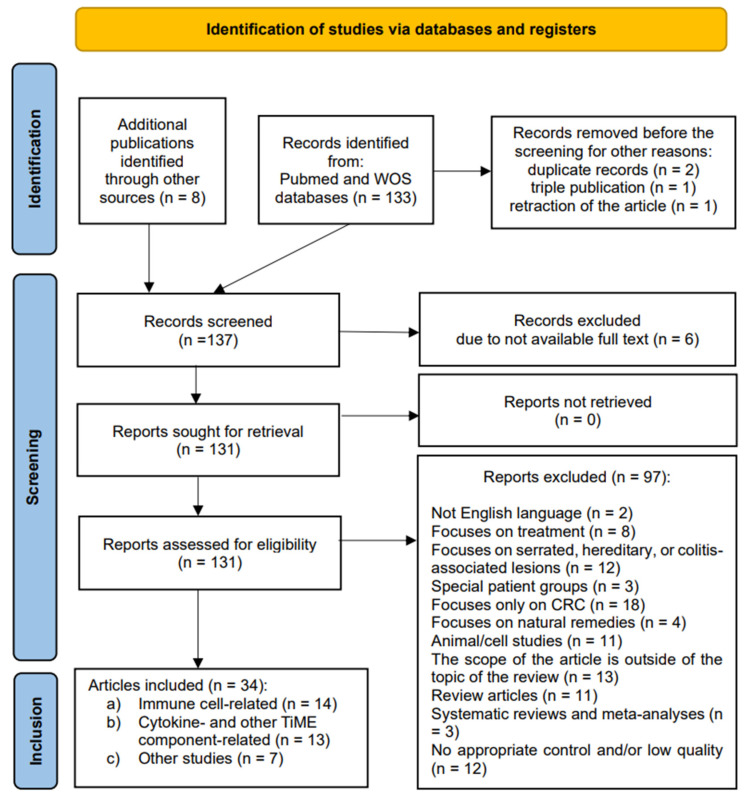
A PRISMA flow diagram indicating the selection of studies for the systematic review.

The initial search identified 133 results (Figure 1). Eight articles were found using external sources. One article was removed before screening due to retraction. Two duplicates and one triple record were also removed. One hundred thirty-seven articles were screened based on their titles and abstracts. Six were excluded due to the full text not being available. Then, all 131 studies were retrieved for full-text evaluation and eligibility. A total of 97 were excluded as ineligible for inclusion. The reasons varied greatly: 2 were in languages other than the English language, 8 focused on treatment, 12 examined pathways other than the conventional carcinogenesis pathway, 3 examined only special patient groups, 18 were limited to malignant CR lesions, 4 highlighted effects of natural remedies, 11 were animal- or cell-based studies, 14 were deemed to be excluded as various reviews and meta-analyses, 12 had no appropriate control and/or were of low quality, and, finally, 13 fell out of the scope of the review due to other reasons. All the studies included were observational: cohort, cross-sectional, and case-control studies. No randomized control trials were identified. A total of 34 studies fulfilled the inclusion criteria and were finally selected for a qualitative analysis.

The studies included were grouped according to the component of the TiME examined for alterations of local immune response in patients with sporadic CR adenoma: (a) studies investigating the cellular component changes (n = 14), (b) studies examining the cytokine- and/or other TiME-component-related immunological patterns (n = 13), and (c) other relevant and novel research in the field, though lacking an appropriate control group (n = 7).

Most of the studies included used the same ‘immune infiltration’ or ‘immune infiltrates” terms for describing the immune cell composition in the immunological milieu, both in the normal CR tissue and the sporadic colorectal neoplasia [50,51,52,53,54,55,56,57,58,59,60,61,62,63], while the terms ‘local gut immunity’ or ‘(tumor) immune microenvironment’ were used for broader compositional and functional evaluation of cell-, cytokine-, and/or other immunological factors in the normal/healthy CR tissue and adenomatous/cancerous tissue specimens, respectively [64,65,66,67,68,69,70,71,72,73,74,75,76,77,78,79,80,81,82,83]. Several studies referred to the ‘immunome’ [54,62,63,70,83] as the genetic background of prevailing immune landscapes. The term ‘immunophenotype’ was employed in the whole majority of the studies describing the antibody-based identification of dominant cellular components in the TiME. Similarly, the term ‘(local) immune system response’ was utilized to illustrate the changes in immune patterns and their trends of increasing or decreasing observed in the gut mucosa samples of patients with CR lesions along the A–C sequence.

Among the studies included, the composition of immune infiltrates and its shifts throughout the conventional colorectal tumorigenesis was examined in conventional CR adenoma without further specification of the type [51,53,57,59,60,65,66,67,68,69,70,71,72,73,75,80] or sporadic CR adenoma classified by morphology: as tubular, tubulovillous, and villous [50,62,77,78,81] or polypoid and nonpolypoid [58] and/or by the grade of dysplasia: as low grade and high grade [54,55,56,61,74,76,77,78,79,81,83]. Very few trials classified adenomas according to size as small and large [58] and according to the location in the gut as proximal and distal [81,82]. Only one study examined the immune infiltration in advanced adenomas [63]. Despite focusing only on the sporadic adenoma stage, two trials [52,55] have also assessed local immune infiltrates in microadenoma, described as the aberrant crypt foci. The latter is treated as the initial preneoplastic lesion, possibly leading to adenoma and further stages of the conventional A–C pathway. Moreover, two studies included the stage of intramucosal carcinoma (carcinoma in adenoma/carcinoma in situ), which follows the advanced adenoma during its stepwise progression into invasive CRC [56,77].

All the studies included in the systematic review examined patients with CR adenoma and invasive carcinoma (in exposure groups) compared to healthy patients’ CR mucosa specimens [51,52,53,55,57,63,65,66,67,69,71,72,73,75,76] or (adjacent) normal mucosa samples of patients with dysplastic lesions [50,54,56,59,60,61,62,64,70,74] (in control groups). A double control group consisting of normal mucosa and healthy patients’ CR mucosa samples was employed in two studies [63,68]. A non-neoplastic mucosa was used for a control group in two studies as well [56,80]. Six trials lacked a control group; however, due to high relevancy and novelty in the field, they were not excluded from the systematic review [77,78,79,81,82,83]. These studies were, therefore, precisely assessed for risk of bias and quality and additionally labeled in the analysis for the critical evaluation of results [77,78,79,80,81,82,83].

In exposure groups, the majority of the studies used tissue specimens sampled directly from the CR lesion. However, adjacent-to-small (less than 1 cm in size) adenoma specimens were also described [52,55,58]. These “off-tumor” samples were obtained to avoid hindering normal pathological examination due to potentially insufficient material after sampling the small lesions directly.

Although all the studies focused on conventional CR carcinogenesis, serrated lesions were also examined in several trials [62,78,81], providing data on local immunity throughout the alternative pathway. An additional hyperplastic polyp exposure group was included in a large number of studies as well [51,56,64,76,77,78,83]. One study examined ulcerative colitis-associated lesions [70], one incorporated a general cohort on inflammatory bowel disease, and two assessed the immune environment changes in FAP lesions [61,79], all in separate exposure groups [52].

Of the studies included in the review, one aimed to specifically compare local immune infiltration in a Norwegian cohort [63], and one compared African Americans and Caucasian Americans in separate ethnicity-based cohorts. The aforementioned facilitated the analysis of significant immunological differences in the premalignant CR lesions according to race and other clinicopathological variables, such as the location, degree of dysplasia, and/or percentage of villous histology [82].

For the assessment of immune cell counts, densities, ratios, distribution between epithelial and stromal compartments, and cytokine and other TiME component expression in CR tissue, twenty-seven studies used an immunohistochemical analysis: simple (IHC), double (double IHC), and/or multiplex (MxIHC) [50,51,52,53,54,56,57,58,59,60,61,62,64,65,66,67,69,70,72,73,74,75,76,77,78,79,83], twelve employed (quantitative) real-time polymerase chain reaction (q)RT-PCR [51,54,57,63,65,67,69,71,72,73,75,79], and nine used immunofluorescence: simple (IF), double (double IF), or multiplex (MxIF) [51,55,62,71,72,73,75,81,82]. Two trials performed Western blot analysis (WBA) [55,70], three used enzyme-linked immunosorbent assay (ELISA) [68,70,75], and one used flow cytometry [68]. Three trials employed H&E staining to quantify eosinophils in CR tissue [77,78,79]. One study incorporated gene set enrichment analysis (GSEA) [58], one used 16S rRNA gene sequencing [80], one used whole-exome sequencing [62], one study used transcriptome RNA sequencing [83], and two used single-cell RNA sequencing [62,67]. Two studies additionally performed a colocalization analysis to examine the spatial distribution and cell–cell interactions between single cells [51,55].

**Figure 2:**
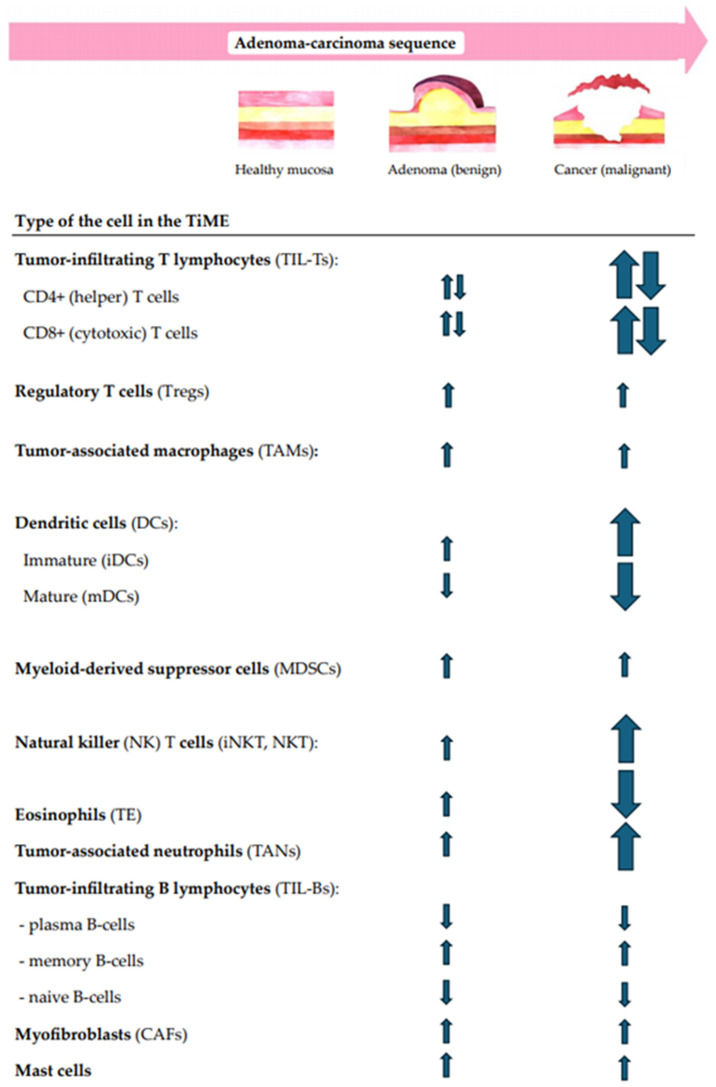
A summary of the main tendencies of immune cellular changes in CR tissues along the conventional ACS.

**Figure 3:**
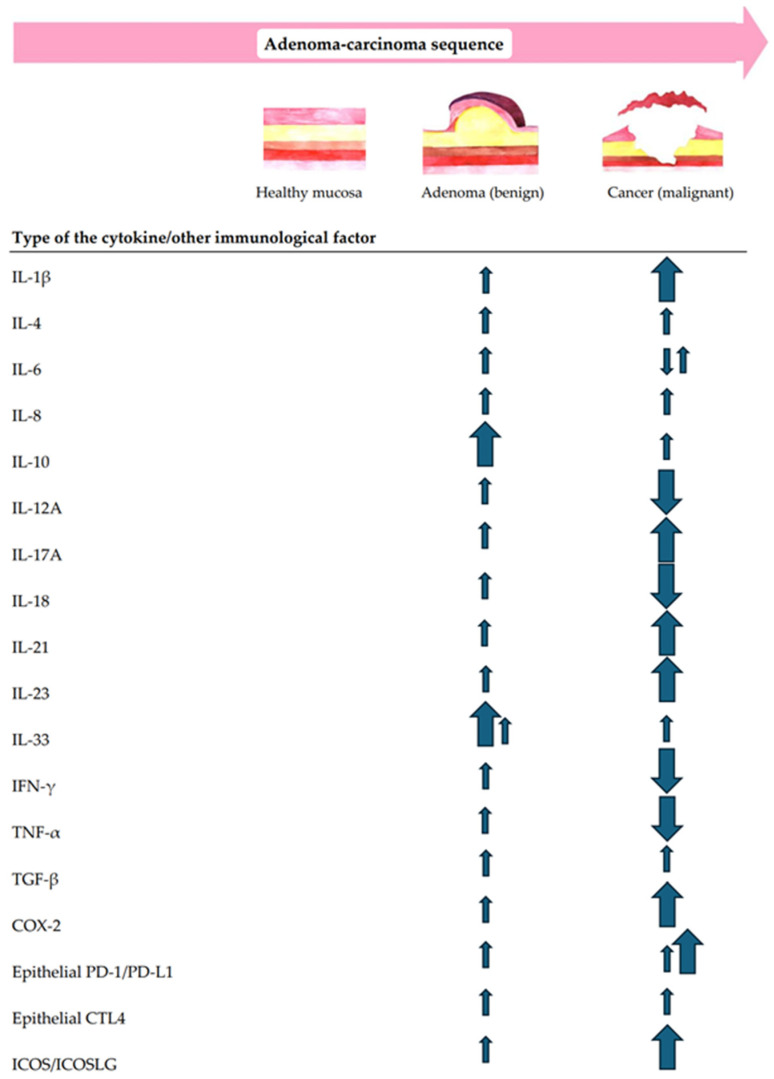
A summary of the main tendencies of cytokine- and/or other immunological factor-related immune changes along the conventional A–C pathway.

In addition to the multidimensional structural analysis of immune infiltrates, the correlation between the local immunity changes and CR lesion morphology, size, dysplasia grade, and location in the gut were questioned. Moreover, the immune shift tendencies for increases or decreases along the ACS were also analyzed. The summary of immune cell-, cytokine-, and other TiME-component-related compositional changes in premalignant versus malignant CR lesions along the conventional A–C pathway are shown in Figure 2 and Figure 3.

Table 2 displays the characteristics and clinical evidence of the studies. Additionally, a Supplementary Materials (Table S3) provides an extended version of the table explicitly describing the composition of immune infiltrates and the alterations in multiple types of immunological factors in sporadic CR adenomatous tissue and cancerous tissue compared to adjacent normal mucosa and/or healthy patients’ CR mucosa specimens.

Eighteen studies were evaluated as representative with an estimated more than 100 subjects in exposure groups. Based on the NOS assessment, 8 studies scored 5/9, 12 studies scored 6/9, and 13 scored 7/9 (Table 1). Overall, high heterogeneity was observed in the study designs, study populations, and the examination methods of the immune infiltration in dysplastic CR lesions along the ACS.

## 4. Discussion

Colorectal cancer represents a diverse group of malignancies that differ not only in pathophysiological mechanisms, therapeutic responses, and clinical prognosis but also in immune response and infiltration [84]. The role of the local immune system in conventional colorectal carcinogenesis remains an emerging field of study. It is known that the immune environment plays a dual role in the development and progression of sporadic colorectal cancer, on the one hand exerting tumor-promoting immunological factors and on the other hand acting tumor suppressively [14,15,16,84]. Despite the emerging evidence in the immune landscapes of invasive CRC, still, little is known about the mechanisms regarding the TiME in sporadic precancerous lesions. Thus, the colorectal adenoma, and in general the conventional colorectal tumorigenesis as the gradual transition from normal mucosa to, eventually, invasive carcinoma, is pending comprehensive analysis regarding its complex signatures of local immune responses and related factors.

To our best knowledge, this systematic review is the first to analyze sporadic premalignant colorectal lesions at once for immune cell-, cytokine-, and other immunological factor-related changes in the TiME along the conventional ACS. In contrast to previous reviews, we focused only on conventional colorectal carcinogenesis. Still, the study was not limited to examining one type of immune component, enabling a holistic analysis of the local immune system’s populational, phenotypical, and functional patterns in health and CR tumorigenesis.

### 4.1. Immune Cell-Related Changes in Patients with Sporadic Colorectal Adenoma (Along the A–C Pathway)

The tumor microenvironment is defined as the location where dysplastic, tumor-associated stromal, and immune cells interact by producing specific cytokines and chemokines while being modulated by various molecular mechanisms and other immunological factors [85]. Here, the complex interaction of cells within the TiME decisively determines whether colorectal premalignant lesions will stabilize or advance.

While the cellular immune landscape of colorectal adenocarcinomas is shown to be highly intricate and prominent, the immune response in colorectal adenomas is thought to be less intense, though in some cases mirroring the immune patterns of invasive lesions [86]. The features of intralesional immune cell populations vary in cell type, count, density, ratio, and cell distribution among epithelial and stromal compartments and possibly depend on lesion morphology, the size, the grade of dysplasia, and the location in the gut. Further, we discuss the significant immune cell-related changes in the TiME of CR lesions along the A–C pathway (Figure 2).

T lymphocytes are the primary immune cells found in precancerous colorectal lesions [87]. There is a notable increase in tumor-infiltrating lymphocytes (TILs) within colorectal adenoma tissues. Here, TILs are predominantly located in the stroma and infiltrate the dysplastic adenomatous epithelium. These TILs comprise CD4+ T helper (Th) cells and CD8+ cytotoxic T lymphocytes [18,88]. Th cells are essential in shaping the host immune response, particularly against cancer. Their function in the TiME is complex and varies based on the specific subsets and conditions. While Th1 cells secrete IL-6, Th17 cells contribute to the secretion of TNF-α, IFN-γ, IL-17, IL-22, and IL-21, and Th2 cells promote intestinal mucosa inflammation by secreting IL-4 [88,89]. Overall, Th1 cells are known to provide protective effects, while Th2 and Th17 cells are linked to tumor promotion and angiogenesis [90].

The data from our systematic review on the CD4+ T cell infiltration in premalignant CR tumor tissues are controversial. While some authors suggested that CD4+ cell concentration increases going through the adenoma–carcinoma sequence [53,54,58,83], others show that CD4+ T cells decrease as adenoma turns to CRC [55,79]. Additional observations regarding microadenomas indicate that Th cell density is similar to that in normal mucosa; however, there is a significant reduction in CD4+ cell infiltration in CRC tissues [55]. There was also a direct association between the changes in Th cell infiltration and premalignant lesion size [54,58] and/or dysplasia grade [58,79]. Moreover, a higher number of infiltrating CD4+ T cells was detected in polypoid compared to nonpolypoid precancerous lesions [58]. Regarding CD4+ T cell distribution among TiME compartments, Th cells were more commonly observed infiltrating the lamina propria and dysplastic adenomatous epithelium [53].

New Th lymphocyte subsets, like Th17 cells, have been identified and studied mainly in premalignant lesions [91]. Our data show an increasing tendency of Th17 cells in CR adenomas and CRC tissues along the conventional ACS [67,68,81]. Our data indicate that proximal colonic lesions had a significantly higher count of Th17 cells than distal colon and rectal adenomas [81].

CD8+ (cytotoxic) T cells are important in eliminating transformed cells by releasing toxic granules upon recognizing specific tumor antigenic peptides presented on the surface of tumor cells [88]. The infiltration and function of CD8+ T cells in the TiME determine resistance to tumorigenesis. The literature declares a higher population of CD8+ cells found in premalignant adenoma tissues than in CRC tissues [92]. Some authors declare a more prominent rise of CD8+ T cells in tumors with ulcerating features than in those with sessile or polypoid appearance [35]. Interestingly, this study revealed that EMAST (elevated microsatellite alterations at selected tetranucleotide repeats)-positive tumors had an increased CD8+ T cell infiltration compared to EMAST-negative tumors in both tumor cell nests and tumor stroma. CD4+ T lymphocytes did not show any correlation with EMAST genomic subtypes.

Across the studies included in our systematic analysis, the levels of cytotoxic CD8+ T lymphocytes vary. More frequently, a decrease in CD8+ T cells was noticed in the adenoma-to-carcinoma transition [56,79]. The opposite opinion also exists, showing that CD8+ T cells increase as the histology of the lesion evolves throughout the normal mucosa–microadenoma–adenoma–carcinoma sequence [55].

Similar to high-grade adenomas, the number of CD8+ T cells in low-grade adenomas was higher in the boundary of the tumor than in the tumor center [56]. Independently of the dysplasia grade, the CD8+ T cells/Tregs ratio was significantly lower in the tumor center of adenoma. CD8+ T cell densities did not increase significantly with lesion size (especially in polypoid lesions) [58], though they were higher in polypoid versus nonpolypoid lesions. This study showed a CD8+/CD4+ cell ratio of 0.4 in small polypoid lesions, 0.3 in large polyploids, and 0.23 in nonpolypoid CR lesions of all sizes [58]. Moreover, the CD8+/CD4+ cell ratio was higher in serrated compared to conventional CR lesions [62].

Regulatory T cells (Tregs) are known for modulating the immune response by infiltrating the TiME and exerting potent immunosuppressive effects that promote tumor progression [18,88,89]. They inhibit the proliferation and cytotoxic function of effector CD8+ and CD4+ T cells by secreting immunosuppressive cytokines, such as TGF-β and IL-10. A decrease in the CD8+ T cell/Treg ratio, therefore, may contribute to the immunosuppressive environment suitable for the progression of adenomas to CRC. Tregs also induce myeloid suppressor cells by interacting with them, leading to a suppressive phenotype that promotes tumor angiogenesis and immune evasion [90]. There are different subpopulations with unique characteristics and functions among Tregs. FOXP3+ Tregs are the most abundant in the colorectum and are associated with a worse prognosis in CRC [93].

Our review shows a gradual increase in FOXP3+ Tregs in CR lesions along the conventional ACS, with a slight rise in colorectal adenoma and a more significant increase in carcinoma tissues [56,57,59,83]. However, FOXP3+ Tregs do not always show a substantial and stepwise increase in normal mucosa–adenoma–carcinoma tissues. This is illustrated by a meaningful rise in FOXP3+ Tregs in colorectal adenoma tissue compared to normal mucosa but only slightly elevated numbers of FOXP3+ Tregs in CRC compared to adenomas [57]. This indicates that the occurrence of high FOXP3+ expression is an early event in the adenoma–carcinoma sequence. One study showed evidence of decreasing numbers of FOXP3+ Tregs throughout the sporadic adenoma–carcinoma sequence [79]. Still, these results should be evaluated critically due to the lack of a control group in the study [79].

Low-grade dysplasia (LGD) levels of FOXP3+ regulatory T cells were found to be higher in sporadic cases rather than FAP cases [61,79], implying that the host has a better tolerance for hereditary lesions than sporadic ones, thus possibly facilitating the earlier occurrence of adenomas and carcinomas in FAP patients. In all three categories—normal tissue, adenoma, and CRC—FOXP3+ Tregs rarely infiltrate the epithelium, with FOXP3+cell infiltration predominantly observed in the lamina propria in healthy tissue, in the adenomatous stroma in premalignant lesions, and in the tumor stroma in CRC [57].

No statistically significant differences were found in FOXP3+ T cell levels between small and large adenomas; however, FOXP3+ Tregs were more abundant in polypoid CR lesions than nonpolypoid CR lesions [58].

In CRC carcinogenesis, macrophages, particularly tumor-associated macrophages (TAMs), contribute to primary inflammation-induced mucosal damage and promote the subsequent transition from inflammation to tumor development [94,95]. At first, they originated as M1 and M2 macrophages—two polarized forms of mononuclear phagocyte during in vitro differentiation, which are now broadly adopted by in vivo studies. These two forms have distinct phenotypic patterns and functional properties. M1 macrophages are known for their proinflammatory and antimicrobial qualities, while M2 macrophages demonstrate anti-inflammatory features and participate in waste and apoptotic cell clearance [18,96,97]. Both macrophage subsets participate in CRC progression with a certain dynamic balance kept between them. In early CRC carcinogenesis, M1 macrophages dominate, but as the tumor progresses, M2 macrophages become dominant. The effects of TAMs in human CRC development and progression are controversial or even contradictory: while some studies declare a high density of macrophages as an indicative factor of a favorable outcome [98,99], other data support the opposite finding [100]. Two antibody markers are most frequently used for the detection of TAMs in performing immunohistochemistry (IHC): the CD68 marker—although being not specific for M1 macrophages, its expression has been upregulated in M1 compared to M2 macrophages—and CD163 marker—highly expressed on the surface of M2 macrophages.

Our study provides evidence that CD68+ cell expression rises along the stages of the adenoma–carcinoma transition [63]. However, a study where TAMs were examined in the low-grade dysplasia (LGD)–high-grade dysplasia (HGD)–invasive carcinoma pathway has shown a rise in CD68+ cells in adenomas, which was followed by a decrease in the invasive carcinoma stage [54]. This study also found a positive correlation between adenoma size and the abundance of macrophages. CD68+ cell and CD163+ cell expression were higher in polypoid than nonpolypoid lesions, regardless of lesion size, and were elevated in large nonpolypoid lesions [58]. CD68+ cells were significantly higher in the sporadic CRC patients than in FAP-diagnosed patients in LGD and stayed similar in both patient groups in HGD [61]. TAMs were found oriented towards the lumen in normal mucosa and hyperplastic polyp tissues, while in colorectal neoplasms along the ACS, TAMs were primarily distributed in the stroma [50]. Similarly, in contrast to serrated lesions, which were located mainly on the luminal surfaces, in conventional adenomas, TAMs were found to be distributed throughout the stroma [62].

Dendritic cells (DCs) are called the most efficient antigen-presenting cells. DCs play a dual role in CRC progression by promoting strong T cell activation to trigger antitumor immune responses while inhibiting tumor-related factors that foster CRC immune tolerance and cancer advancement [101]. DCs are divided into two subsets of mature (mDCs) and immature (iDCs) dendritic cells, which are conversely associated with CR lesions along ACS [102].

Our systematic review has revealed mDC (CD83+, Cd208+) density being slightly decreased in premalignant lesion tissues but significantly decreased in CRC tissues, and the iDCs (CD1α+) density gradually increased from adenoma to carcinoma tissues compared with healthy controls [51]. The same study also examined the distribution of tissue-infiltrating DCs: mDCs were not homogenously distributed in the stroma of CRC and were mainly invading the edges of malignant lesions, while in CR adenoma, mDCs were abundantly distributed in the subepithelial stroma. Increased iDCs were observed in the intratumoral mass, some invading the malignant epithelium [51]. Another study showed that S-100+ DCs are primarily oriented toward the lumen in normal mucosa and hyperplastic polyp tissues, whereas increased S-100+ DC infiltration is confirmed in CR neoplasms of ACS. Here, the DCs are distributed mainly in the stroma of CR lesions [50].

Myeloid-derived suppressor cells (MDSCs) are a heterogeneous population of granulocytes and monocytes that rapidly expand during infection, inflammation, and cancer [103]. MDSCs possess immunosuppressive functions that enable cancer to evade the immune system, facilitating further tumor development [103]. Research indicates that the level of circulating MDSCs rises significantly during the advanced stages of CRC and correlates with disease progression and the development of metastases. However, recent findings by Ma et al. have demonstrated that MDSC levels in circulation can also increase during premalignant conditions, such as colon polyposis [104].

Our systematized data showed a prominent increase in MDSCs in both CR adenoma and CRC [60,75]. Regarding cell distribution, the MDSCs seem to expand to the middle part of the transitional crypt in sporadic premalignant and malignant CR lesions [60].

Natural killer (NK) T cells are potent cytotoxic T lymphocytes that play a crucial role in the innate immune response by eliminating abnormal cells without depending on specific antigens [105]. These cells are classified into types I and II according to their function and activity spectrum. Type I–CD1d-restricted invariant natural killer T (iNKT) cells are early-responding, powerful regulatory cells of immune responses involved in tumor immuno-surveillance [106,107]. Type II NKT cells significantly contribute to intestinal inflammation and colitis. A high infiltration with iNKT cells has been associated with a favorable prognosis in CRC, compared with infiltration with II NKT cells or without NK cell infiltration at all [105,106,107]. Furthermore, the study examining the role of NK cells in early CR carcinogenesis reported that Type I–iNKT cells may contribute to intestinal polyp formation by suppressing Th1 immunity and promoting regulatory T cells in the CR tissue [108].

According to our systematic review, all studies examining the NK cell-related immune landscape [54,55,62,71,81] except for one [79] have shown increased NK cell levels in colorectal adenoma compared to normal mucosa or healthy patients’ controls. This could be possibly explained by the usage of different markers: CD56+ (a marker of a large majority of NK cells, especially early-responding iNKT cells) in the studies that found increased NK cell infiltration [54,55,62,71,81] and CD57+ (which refers to the more mature phenotype of NK cells) in the survey with a tendency for a decrease in the late carcinoma stage [79]. The study on immune cellular changes in tissue with aberrant crypt foci (ACF) along the conventional ACS indicated a slight increase in NK cell infiltration during the microadenoma stage and a more substantial increase in the carcinoma stage, suggesting early and constant NK cell involvement in the conventional normal mucosa–microadenoma–adenoma–carcinoma transition [55]. Higher quantities of NK cells were observed to be more prevalent in serrated compared to conventional CR polyps [62], in proximal rather than distal CR lesions [81], and more commonly associated with tubular than villous histology [81]. Increased NK cell infiltration in the CR adenoma and carcinoma was also related to the over-expression of IL-21, which is known to enhance NK cell activity [71]. This study also discovered that NK cells are mostly located in the tumor stroma of dysplastic CR lesions [71].

To date, the accumulation of tissue eosinophils (TE) in the CR mucosa is known for shaping the local microenvironment, which in turn impacts the development and progression of CRC [109]. In invasive CR carcinoma, eosinophils are located both in the center of the tumor and in front of the invasion, suggesting an active role in the TiME [110]. Interestingly, tissue eosinophilia is a favorable prognostic factor independent of the tumor stage, histological grading, and vascular invasion [111]. However, this aspect is often overlooked in routine clinical practice. The literature declares that the counts of tumor-infiltrating eosinophils increase in premalignant colorectal lesions [112] and decrease as the adenoma development progresses [110,112,113]. In tubular adenomas with LGD, a notable presence of infiltrating eosinophils is observed, whereas fewer eosinophils are noted in cases of adenomas with HGD [112]. Adenocarcinoma cases are believed to show only minimal eosinophil infiltration [112].

Only two studies on TE in premalignant CR lesions were found to fulfill the inclusion criteria and meet the quality requirements for our systematic review [77,78]. A substantial decrease in TE was found as the malignancy potential increased [78]. However, there was no statistically significant difference in the degree of TE between cases of LGD and HGD [78]. While higher quantities of TE were observed in the malignant lesion’s transitional zone (between normal tissue and carcinoma), it decreased in the cancerous stromal region [78]. Another study reported an inverse relationship between carcinoma’s invasiveness and stromal TE intensity [77]. Therefore, TE, in general, was noticed as a promising marker in CRC formation and could become a diagnostic for polyp differentiation as well as serve as a favorable prognostic marker in CRC [109,110].

Increasing evidence highlights the role of neutrophils in the context of CRC. Tumor-associated neutrophils (TANs) participate in the transformation of inflammation into CRC, referring to inflammation-linked (e.g., inflammatory bowel disease (IBD)-associated) tumorigenesis [114]. However, accumulating data have also reported increased TAN infiltration and neutrophil/lymphocyte ratio (NLR) across the stages of the conventional A–C pathway [115]. In adenomas, the NLR is associated with polypoid morphology and polyp size, and patients with polyps larger than 10 mm tend to have significantly higher NLRs than those with polyps smaller than 10 mm [42]. Another study revealed that the leukocyte count, neutrophil ratio, and NLR are highest in CRC and diminish gradually backward in the ACS [116]. Thus, NLR is postulated to have predictive significance in distinguishing the neoplastic potential of colonic polyps and could be used for monitoring polyps [116].

The study [52] included in our systematic review revealed that myeloperoxidase-positive (MPO+) cells (referring to neutrophils and monocytes) gradually increase along the ACS. Myeloperoxidase (MPO) is one of the main enzymes released in neutrophil activation. An increasing MPO+ cell pattern has been shown to rise from normal mucosa to carcinoma progressively [52]. The mean number of MPO+ cells was found to be similar in both ACF and adenomas, indicating no significant difference between the two. The average number of MPO+ cells was found to be higher in dysplastic ACF compared to nondysplastic ACF. Collectively, these findings suggest that MPO immunohistochemistry is a simple method for detecting inflammation in CR mucosa and could potentially be used to assess the risk of CRC. Moreover, MPO+ cells were more abundant in MSI (microsatellite instable) versus MSS (microsatellite stable) colorectal carcinomas, questioning MSI’s influence on host immune responses to CRC [52]. Another study has shown increased counts of TANs in colorectal adenomas compared to normal mucosal tissue. In addition, these counts were positively correlated with dysplastic lesion size [54].

An opposing trend was observed regarding tissue monocytic infiltration, showing a progressive decrease in monocyte counts from advanced adenoma (AA) to invasive CRC stages [63]. This was, additionally, followed by an increase in TAMs in the TiME of AA and CRC [63]. The latter is fully explainable by the well-known monocytic migration to the tissue and differentiation into tissue macrophages and/or dendritic cells upon tissue damage or inflammation [117].

Tumor-infiltrating B lymphocytes (TIL-Bs) are a heterogeneous subgroup of adaptive immune cells in the TiME, directly and indirectly exerting anti- and pro-tumor effects through antigen presentation and antibody and/or cytokine production [118].

Our data state that L26+ B cells in the CR adenoma-to-carcinoma tissue are found to reside in the epithelial compartment of lymphoid follicles [50]. A more detailed composition of TIL-Bs was described by a decrease in plasma and naive B cell counts and by an increase in memory B cells [63] in the TiME along the conventional ACS.

The TiME also consists of a stromal compartment where myofibroblasts (or myofibroblastic cancer-associated fibroblasts—CAFs) reside. Originally gastrointestinal stromal cells, myofibroblasts are markedly upregulated in CRC, suggesting a potential functional role in CR tumorigenesis [119]. Myofibroblasts are believed to evade apoptosis and remain hyper-activated, secreting elevated amounts of extracellular matrix molecules, cytokines, and matrix-degrading enzymes during this stepwise process. Additionally, they affect the immune response, induce pro-invasive signals for tumor cells, and influence epithelial-mesenchymal transitions [119,120].

The studies in our systematic review regarding myofibroblast-associated changes in CR premalignant lesions were not entirely conclusive. The increased density of myofibroblasts was associated with over-expressed IL-8 levels [66]. The higher counts of myofibroblasts were found within the lamina propria [53] and/or the stromal compartments [73] of CR adenoma and CRC.

It is noteworthy to mention mast cells, which in the TiME act as the barrier between the host and the outside environment. In addition to their other functions, these cells can secrete cytokines that alter tumor growth in the inflammatory milieu [121,122]. Mast cell proliferation in CRC is associated with angiogenesis, the number of lymph nodes to which the malignancy has spread, and patient prognosis [121].

Our systematized data showed increased levels of mast cells activated in AA and CRC tissues versus normal mucosa and healthy patients’ tissues [63]. Higher counts of CD117+ mast cells were positively correlated with the proximal colon compared to distal CR dysplastic lesions and inversely related to the villous component of the lesion [81]. Interestingly, mast cells were less abundant in CR adenomas of African Americans rather than in Caucasian Americans [82]. However, the latter should be interpreted only as an observational finding due to the lack of a control group and precise descriptions of the cohorts in this study.

### 4.2. Cytokine-Related Immune Responses in Patients with Sporadic CR Adenoma

Along the ACS, not only cells but also cytokine activity matter. In fact, malignant transformation involves a balance of pro-tumorigenic and antitumorigenic cytokines, which is essential for maintaining homeostasis [123]. Once this balance is disrupted, the tumor immune surveillance escape occurs [124]. Clarifying the role of not only cellular but also inflammatory and immunomodulatory cytokine pathways is mandatory for an integral understanding of CR lesion-associated immune patterns along the adenoma–carcinoma transition. The specific roles of cytokines in malignant CR lesions have been thoroughly explored and documented in the literature [123,124,125]. Therefore, further, we discuss the most highlighting tendencies of cytokine-related immune changes in patients with colorectal adenoma in the context of conventional CR carcinogenesis (Figure 3).

Our results showed that cytokine levels change significantly along the conventional CR dysplastic lesion transformation [54,57,59,65,66,67,68,69,70,71,72,73,74,75].

Interleukin-1 beta (IL-1β) is an IL-1 family proinflammatory cytokine secreted by immune, stem, and tumor cells [123]. Its upregulation is closely associated with various cancers, including CRC [124,125]. Our data found that IL-1β expression gradually increases from a slight rise in adenoma to a more substantial over-expression in carcinoma tissues [68,75]. Additionally, it was observed that IL-1β activates the expression of interleukin-8 (IL-8) [75] and stimulates Th17 cells [68] in the analyzed studies. This is consistent with the literature data, stating that IL-1β induces a proinflammatory response by stimulating the expression of TNFα, IL-6, IL-8, IL-17, COX-2, and PGE2 [123].

Our data report that the levels of Th2 cytokines—interleukins-4 and -10 (IL-4 and IL-10)—gradually increase from CR adenoma to CRC. The over-expression of IL-4 and IL-10 in tissues was increased in CR adenomas versus healthy subjects and was only non-statistically higher in CRC than CR adenomas [65]. The early expressional changes in Th2 cytokines may suggest their role in the earliest stages of CRC development. This study has also revealed that cytokine-expressing cells are polarized to the subepithelial stroma in adenomas and distribute evenly through the stroma in CRC [65].

The exact pathway of interleukin-6 (IL-6), a multifunctional cytokine expressed in CR adenomas, has not yet been discovered. The IL-6 expression level elevates gradually during the progression from colorectal adenoma to carcinoma [124,126]. A clear correlation between IL-6 concentration and the risk of polyp number or its type is not yet proven. Instead, IL-6 was admitted to shift the Th1/Th2 balance towards Th2 cells [126].

Data from our review on IL-6 levels in precancerous and cancerous tissues has been contradictory. While one trial reported decreasing expression of IL-6 cytokines in CRC samples vs. normal mucosa and healthy patients’ control specimens [68], the other two studies showed over-expression of IL-6 genes in cancerous CR tissues compared to healthy controls [63,81]. The rise of IL-6 levels was even higher in advanced adenomas than in invasive carcinomas [63]. No significant change in IL-6 levels was observed in lesions with LGD compared to those with HGD [81]. However, the presence of a villous component significantly influenced IL-6 expression in colorectal lesions. Specifically, lesions with a higher villous component exhibited lower IL-6 levels than those with a lower one [81]. A decline in IL-6 expression was observed from proximal colonic adenomas to adenomas located in the left-sided colon and/or rectum [81].

The activated interleukin-8 (IL-8) network in the TiME may serve as a key regulatory factor for the progression of CR adenoma and the A–C transition. The review data showed a gradual increase in IL-8 mRNA levels in adenoma and carcinoma [54,66,74,75]. Concerning the distribution of IL-8, the cytokine and its receptors (IL-8RA, IL-8RB) were observed in the stroma of both adenomatous and cancerous cells [66]. The co-expression of IL-8RA and IL-8RB with CD34+ tumor-associated microvessels in both the adenoma and CRC [66] or with LGR5 labeled cancer stem-like cells (CSCs) in CRC tissue sections [75]. The over-expression of IL-8, together with TSP50 and SERCA2, was statistically significantly associated with the degree of dysplasia in CR adenoma as well as with the lympho-vascular invasion, advanced TNM staging, and high intra-tumoral inflammatory infiltrate in CRC, overall with worse prognosis [74].

Regarding IL-10 levels in premalignant and malignant CR lesions, double immunohistochemistry revealed more abundant CD4/CD25 and IL-10/FoxP3 dual-positive Tregs within the tumor stroma in both adenomas and carcinomas [57]. The findings imply that the over-expression of IL-10 may be partially attributed to the increased presence of Tregs in CR adenoma during the transition to CRC. These elevated levels of Tregs are likely responsible for the release of some IL-10. In addition, IL-10 was expressed in the tumor stromal cells and epithelial cells of both premalignant and malignant CR lesions [57]. The density analysis disclosed that IL-10 levels are significantly higher in adenoma tissues than in the normal control and slightly higher than in CRC [57]. Another trial [59] reported the trend of gradually rising IL-10 levels along the ACS, showing its positive correlation with the histological grade and TNM stage in cancerous CR lesions [59].

Interleukin-12A (IL-12A), interleukin-18 (IL-18), interferon-gamma (IFN-γ), and tumor necrosis factor-alpha (TNF-α) are classified as Th1 cytokines in the TiME of (pre)malignant CR lesions [123,124,125]. The literature declares that IL-12A and IL-18 act synergistically: IL-12 upregulates the expression of the IL-18 receptor on cells producing IFN-γ [127]. Together with TNF-α, they are over-expressed in premalignant adenomatous lesions and diminish during the progression to the malignant stage of CRC [128].

Similarly, our systematic review showed that Th1 cytokines (L-12A, IL-18, IFN-γ, and TNF-α) are upregulated in CR adenomas but turn downwards in CRC tissues [65].

IFN-γ, known for its extraordinary role in initiating antitumor immunity and immunoediting [129], was also noticed to decrease in distal rather than proximal CR lesions and in adenomas with villous rather than tubular histology [81].

Considering the Th1/Th2 cytokine ratio and its significance along the conventional ACS, the rise of Th1 cytokines in CR adenomas probably signals a proactive host response to mutated cells, reflecting an initial effort to combat the tumor [125,126,127]. However, in CRC, there is a notable decline in Th1 cytokine gene expression within the TiME, replaced by Th2 cytokines. This shift indicates a reduction in the local antitumor immune response observed in the invasive cancer stage.

The interleukin-17 (IL-17) family comprises a group of proinflammatory cytokines, including six homologous proteins (IL-17A to IL-17F). Most studies indicate that IL-17A plays a pro-tumorigenic role, as it is over-expressed throughout all stages of the A–C pathway [123]. IL-17A is associated with varying degrees of dysplasia [130]. Its expression levels are higher in CRC compared to earlier stages of the disease progression. Additionally, IL-17 contributes to establishing precancerous CR adenomas [130].

Our data declare that the IL-17A expression level in both CR adenomas and CRC increases continuously from the adenoma stage to the CRC stage [67,68,70,73]. The IL-17A-secreted cells were found in both the lesion’s stroma and epithelium [67] and were stimulated by IL-1β, IL-6, and TGF-β in the progression of CRC [68]. Interestingly, elevated levels of IL-17A were not associated with the severity of dysplasia, showing similar expression in low-grade and high-grade CR lesions [81]. Moreover, the elevated levels of IL-17A were inversely related to the amount of the lesion’s villous component [81]. Overall, it seems that the activated Th17/IL-17A network in the TiME is significantly associated with a dynamic stromal cellular response throughout the ACS, which might provide a supportive environment for the initiation and progression of CRC.

Interleukin-21 (IL-21) is an IL-2 family cytokine secreted by activated T cells, regulating immune responses [123]. Accumulating evidence suggests that IL-21 plays a significant role in enhancing the immune response by controlling the proliferation of CD4+ cells, the production of immunoglobulin, preventing CD8+ T cell apoptosis, promoting the differentiation of CD8+ cells, and limiting the differentiation of Tregs [131].

Our data showed a stepwise increase in IL-21 mRNA expression, which began to increase at the adenoma stage and was maintained at a higher level at the CRC stage [68,71]. This increase was significantly correlated with longer overall survival times in the CRC patient group [71]. IL-21+ cells were mostly NK cells and T lymphocytes in the tumor stroma [71]. The IL-21 receptor is primarily located in the stroma rather than in the adenomatous dysplastic epithelium, which suggests its role in adenoma transformation may be mainly through regulating immune cell function [87].

Interleukin-23 (IL-23), a proinflammatory factor, has also been shown to participate in the malignant transformation of the colorectal epithelium. It was reported to play a paramount role in the mucosal immune system, as the IL-23 receptor (IL-23R) is expressed specifically in colorectal carcinoma (epithelial) cells [132]. Our analysis revealed gradually increasing levels of IL-23, slightly rising from the premalignant adenoma to a substantial increase in malignant CRC [68].

Recently, the role of interleukin-33 (IL-33) in the pathogenesis of CRC has been discussed [133]. Previously thought to act only pro-carcinogenically, recent findings have revealed a more complex dual role. When expressed in tumor cells, IL-33 enhances the immune response and stimulates type 1 antitumor immunity by activating CD8+ T cells and NK cells. In contrast, when present in the tumor stroma and serum, IL-33 facilitates immune suppression through Tregs and myeloid-derived suppressor cells [134]. In addition, new evidence suggests that IL-33 is able to influence tumor development via eosinophil activation [133].

Our systematized data found that the expression levels of IL-33 and its functional receptor ST2 mRNA were increased in CRC tissues vs. healthy subjects’ samples and were even higher in CR adenomatous compared to cancerous tissues [69]. These shifts were detected in both the tumor stromal cells and dysplastic epithelial cells of the adenoma/carcinoma. Moreover, the over-expression of ST2 in CRC was associated with the TNM stage. Furthermore, increased levels of IL-33+ and ST2+ microvessels were found in the stroma of A and CRC, suggesting the role of the IL-33/ST2 axis in the CR neoplastic transformation by contributing to the regulation of angiogenesis [69]. Another study showed, equally in adenoma and carcinoma, increased densities of ST2-positive cells associated with Treg accumulation within the TiME [72]. This finding implies that the IL-33/ST2 pathway might potentially contribute to immunosuppressive milieu formation during CR carcinogenesis.

Transforming growth factor-beta (TGF-β) dynamics in CR carcinogenesis are also examined [135]. In the early stages of the ACS, TGF-β protein has a suppressive role by inducing cell cycle arrest and apoptosis in the initial stages of tumor development. During cancer progression, tumor cells gradually become resistant and secrete TGF-β themselves, working, in turn, as immunosuppressors facilitating neo-angiogenesis and tumor invasion and metastasis [123,135]. In the TiME, TGF-β expression shows a stepwise increase from normal epithelium to polyp and tumor cells [136].

From the studies included in our systematic review, one study has analyzed the TGF-β expression in premalignant and malignant CR lesions, reporting a gradual increase in TGF-β expression levels and pointing at TGF-β regulation mode in the progression of CRC [68].

### 4.3. Other Immunological Factors in the Progression of Colorectal Adenoma

Immunological factors, such as COX-2, PD-1/PD-L1, CTLA-4, and ICOS/ICOSLG, have also been shown to directly and indirectly impact CR tumorigenesis. Their tendencies for an increase in the TiME of CR lesions going through the A–C transition are displayed in Figure 3.

Cyclooxigenase-2 (COX-2) and its product, prostaglandin E2 (PGE2), have been closely linked to CRC occurrence, progression, and prognosis [137]. The over-expression of COX-2 is found in most colorectal adenocarcinomas compared to adjacent histologically normal mucosa [138]. However, the results on the levels of COX-2 in precancerous CR lesions contradict each other.

Four studies included in our systematic review have reported an elevated expression of COX-2: a slight increase in CR adenomas and a boost in CRC [51,53,56,64]. COX-2 was expressed mostly in tumor lamina propria [53]. In sporadic adenomas, COX-2 expression was found in subepithelial intestinal SMA+ myofibroblasts, indicating them as key target cells for NSAID-mediated chemoprevention of CRC [65]. A study [56] found that the count of Tregs correlates with COX-2 expression. The level of Tregs was notably elevated in tissues displaying COX-2 expression compared to those lacking it, indicating an increasing expression of COX-2 throughout adenoma development.

Programmed cell death 1 (PD-1) and its ligand (PD-L1) participate in modulating the immune response to cancer [139]. PD-L1 expression has been observed in both tumor and immune cells within the TiME of CRC [139]. They are key suppressors of the cytotoxic immune response, nowadays broadly examined as novel targets for oncological immune therapies [140].

Our systematic analysis revealed high PD-L1 epithelial expression in CRC and adenoma with HGD versus normal mucosa specimens [76,80]. PD-L1 expression in epithelial cells was more pronounced in CRC samples than in adenoma with HGD. Similar results proving the increase in PD-L1 expression within the intraepithelial lymphocytes (IELs) through the A–C pathway were provided by another study [83]. PD-L1 expression demonstrates an upward trend, with 6.8%, 37.9%, and 42.0% of high PD-L1 expression in LGD, HGD adenomas, and CRC, respectively. A similar trend was noticed with PD-1 expression as well [83]. In LGD, HGD, and CRC samples, PD-1 was highly expressed in 15.9%, 17.2%, and 48.0% of samples, respectively. In contrast to increasing epithelial PD-1/PD-L1 expression, when measured in tumor stroma cells, it decreases in both FAP and sporadic lesions along the ACS [79]. Prolonged overall survival was observed to have a connection with a low PD-L1 epithelial immunoreactivity score (IRS) and high PD-L1+ TILs IRS [80]. High PD-L1 epithelial IRS is associated with short recurrence-free survival.

It is well-known that cytotoxic T-lymphocyte-associated antigen 4 (CTLA-4) plays a crucial role in the regulation of T cell activation [141]. The data from our systematic review showed a notable rise in CTLA-4 epithelial expression in adenomas with LGD, HGD, and CRC when compared to normal specimens [80], whereas the abundance of CTLA-4+ TILs significantly favored adenomas with LGD over normal mucosa, HGD, and adenocarcinoma cases. A high CTLA-4 epithelial score was linked to positive lymph nodes (LNs), the presence of infiltrative tumor border configuration (TBC), and the absence of peritumoral lymphocytes [80]. Conversely, low levels of CTLA-4+ tumor-infiltrating lymphocytes (TILs) were significantly associated with a more advanced tumor stage and a higher number of positive LNs. Prolonged overall survival correlated with a low CTLA-4 epithelial intensity score and a high CTLA-4+ TILs histoscore. Furthermore, a significant connection was noticed between a shorter recurrence-free survival and a high CTLA-4 epithelial intensity score [80].

Overall, the expression of PD-L1 and CTLA-4 by tumor cells in CRC may collaborate to enhance tumor progression, resulting in poorer patient outcomes. However, their expression by TILs could oppose tumor progression [80].

Inducible T cell co-stimulator (ICOS) and its ligand (ICOSLG) signaling are examined in the TiME of CR lesions as well. ICOS, which was originally identified as a marker of T cell activation, has also been found to play important roles in T cell proliferation and cytokine secretion [142]. ICOS interaction with its ligand, ICOSLG, constitutes a costimulatory signal, inducing the production of a wide range of both pro- and anti-inflammatory cytokines [76].

In the studies included in the review, the expression of ICOS/ICOSLG was found to gradually increase going through the A–C pathway [76]. Increased expression of ICOS/ICOSLG was linked to the progressive formation of FOXP3+ TILs in the TiME, potentially facilitating the transition from precancerous lesions to CRC. Additionally, the elevated co-expression of PD-1/ICOS+ or PD-1/ICOSLG+ contributed to an active TiME along the ACS. PD-1, together with ICOS/ICOSLG expression status, categorized patients with CR lesions into low, moderate, and high risk for progression [76]. The latter may be used in the future to stratify patients with premalignant colorectal lesions, enabling the application of optimal treatment strategies based on the risk of cancerous transformation.

### 4.4. Association with Polyp Morphology, Size, Dysplasia Grade, and Location in the Gut

The systematic analysis has shown that the CR adenoma microenvironment contains a high density of immune cells (compared to healthy controls), exerting cytokines and other immune-related factors of both antitumor and pro-tumor functions. Other factors, particularly those related to morphology, size, dysplasia grade, and position of colorectal adenoma, can also impact changes in the local immune profile.

Immune infiltrates were generally more abundant in preinvasive proximal colon lesions than in the distal colon and rectum [81,82]. This might be because the mucosa in the proximal colon is more porous, and a more efficient bacterial translocation can occur. Therefore, more bacterial antigens are present in proximal colonic lesions, which leads to higher immune infiltration. Moreover, the terminal ileum—a deck of immune cells—is much closer to the proximal colon, allowing for faster immune cell migration to this area [143].

Colorectal lesions, in general, were more commonly found on the left side of the colon than on the right [83]. This tendency can be seen in all types of examined A–C pathway lesions (LGD, HGD, CRC) and is assumably associated with smaller immune infiltrates, less intense immune responses, and longer direct host–microbiota interactions on the left side of the colon and rectum [144].

The expression of most immune markers (CD8+, FOXP3+, CD68+, CD163+, and MHC-I+) was significantly higher in polypoid than in nonpolypoid CR lesions independently of lesion size [58]. Only CD4+ cells did not fit the rule since a significant increase was observed only in large polypoid tumors compared to nonpolypoid counterparts.

Systematized data have revealed that densities of MPO+ cells [52], TAMs [54,61], Tregs [61], and CD4+ T cells [61] in the TiME of premalignant CR lesions were directly associated with the grade of dysplasia. However, tissue eosinophils [78] and mast cells, together with NK cells, IL-6, IL-17A, and IFN- γ [81], were similarly expressed in both LGD and HGD CR lesions.

Lesions with a highly expressed villous histology had lower levels of IFN-γ and IL-6 than lesions with a low percentage of the villous component [81]. This implies that a more aggressive morphological architecture is associated with an immunologically suppressive tumor environment.

Several studies included in the systematic review addressed the changes in immune patterns to the CR lesion size [54,58]. Herein, the densities of TAMs, TANs, activated T cells [54], and CD4+ T cells [58] in the TiME of colorectal adenoma were positively correlated with the polyp size. Conversely, the rise of CD8+, FOXP3+, CD68+, CD163+, and MHC-I+ cell densities was independent of lesion size [58]. The size and villous architecture are widely understood as the aggressiveness of the lesion. However, the theory “the more aggressive lesion, the “colder” immune environment” partially conflicts with the results of our review.

Overall, our systematic review revealed that various immune cells, proinflammatory cytokines, and other immunological factors undergo substantial phenotypic, structural, functional, and gene expression-related changes in the TiME of premalignant CR lesions. These alterations of the TiME may play a significant role in contributing to the progression of precancerous CR lesions to CRC.

### 4.5. Limitations of the Review

All the studies included in the systematic review were comparative observational, as we have not found any randomized controlled trials that would be eligible and evaluated as high or good quality for inclusion. We did not include non-English trials as well. The latter is due to resource constraints and a lack of policy relevance outside English-speaking countries. The abovementioned could have hindered the efforts to avoid selection bias in the review. Moreover, we have not included any experimental cell studies and studies on animals, as we aimed to reveal precisely human-specific immune signatures in the uncontrolled setting of early CR tumorigenesis. Several topic-relevant studies, however, without control groups, were not excluded from the review, though they were additionally marked and isolated, so the results could be interpreted with special caution.

This systematic review was initially designed to analyze immune patterns in colorectal adenomas, the primary precursors of sporadic CRC. However, at the same time, it is limited in its ability to provide a broader comparison of unique immune characteristics among other origin colorectal lesions and different pathogenetic mechanisms, such as the serrated pathway and IBD-associated colorectal carcinogenesis.

Considering a small number of studies examining only the conventional colorectal adenoma-associated immune response, we have included research looking at both sporadic premalignant and malignant dysplastic lesions in the colorectum. This should be assumed as an additional strength of the study, as an analysis of local immunity in every step of the normal mucosa–colorectal adenoma–colorectal carcinoma transition is of crucial importance. Nevertheless, the results of the review could also be hampered by different sample sizes; different study populations (according to age, gender, diet, body mass index, geographic location, and environmental and behavioral factors); various types of controls (adjacent normal mucosa or healthy patient controls); and different assays for the identification of immunocompetent cells, proinflammatory cytokines, and other immunological factors in the gut tissue specimens. Such variability in methodologies used across the original manuscripts could have hindered a more meaningful interpretation of the results.

The outcomes between the trials, including alterations in the immune cell-related and cytokine-associated immune microenvironment in patients with sporadic precancerous +/− cancerous colorectal lesions, were inconsistent and to some extent incomparable. In some cases, the results have shown opposite tendencies in tissue-related immune infiltration. Therefore, a larger sample size and good quality studies (always with an appropriate control group) analyzing the whole spectrum of tissue-associated immune cells, cytokines, chemokines, and immune-related gene expression as well as immune–microbiome–genetic interactions and the impact on diagnostic and therapeutic innovations among different CRC pathogenetic mechanisms are necessary in the future.

## 5. Conclusions

The role of immune cells in CRC progression remains an emerging field of study. This systematic review suggests that precancerous dysplastic lesions in the colorectum show a strong immune response, during which the TiME undergoes marked changes in the counts, densities, and distributions of immunocompetent cells; the expression of immune-related cytokines; and other immunological factors. Moreover, current systematized data advocate that altered immunological parameters may have direct clinical significance at the early colorectal adenoma stage by inhibiting immunosurveillance, preserving an immunosuppressive functional status, and consequently determining the progression of the premalignant lesion to CRC.

Ultimately, the findings of this systematic review demonstrate that colorectal adenoma and colorectal cancer are associated with progressively increased quantities of Tregs TAMs, iDCs, MDSCs, NK cells, TANs, memory B cells, myofibroblasts, and mast cells in the TiME, suggesting their early involvement in CR carcinogenesis. No consensus on the immune-related tendencies concerning CD4+T cells and CD8+ TILs was reached. Decreasing expression of mDCs and plasma and naïve B cells were detected along the ACS. The increased relative density of tissue eosinophils in the adenoma tissue was reported to drastically diminish after the transition to carcinoma. As the adenoma progresses, the increasing expression of IL-1α, IL-4, IL-6, IL-8, IL-10, IL-17A, IL-21, IL-23, IL-33, and TGF-β and decreasing levels of IL-12A, IL-18, IFN—**γ**, and TNFα in the invasive carcinoma stage is being detected. The over-expression of several specific immunological factors, such as COX-2, PD-1/PD-L1, CTLA-4, and ICOS/ICOSLG in the colorectal adenomatous and cancerous tissues, were also observed.

Proximal colonic, polypoid, conditions without expressed villous histology, and in some cases HGD CR adenomas were independently associated with increasing immune response. Still, due to high heterogeneity in methodologies, sample sizes, and controls among the studies included, the results should be interpreted with caution.

Therefore, we urge further studies on the immunosurveillance–immunosuppression status along the adenoma–carcinoma transition, primarily targeting populational, phenotypical, functional, and gene-expression alterations in the immune microenvironment of the premalignant colorectal lesion, as the main precursor of sporadic CRC. Future research is also needed to better understand the role of risk factors (such as sex, age, diet, alcohol consumption, and smoking), gut microbiome involvement, and (epi)genetic changes regarding local immune signatures in patients with dysplastic CR lesions. Understanding the pathogenetic mechanisms involved in the crosstalk between immunocompetent and CR neoplasia cells in the TiME would certainly contribute to improving the existing knowledge of the immunogenic properties of the CRC and could have a substantial impact on the development of novel prevention, diagnostic, prognostic, and therapeutic targets.

## Figures and Tables

**Table 1:** A quality assessment of the selected studies according to the star score of the Newcastle–Ottawa Scale (NOS), based on * which are assigned to three criteria, i.e., the selection (with a maximum of 4 stars [****]), comparability between the case and controls (with a maximum of 2 stars [**]), and the ascertainment of effects of microbiota—outcome/exposure (with a maximum of 3 stars [***]) for a potential score ranging from 0 to 9 points. Higher scores indicate a lower risk of bias.

Author	Selection	Comparability	Outcome/ Exposure	Total Score
Immune cell-related studies				
Banner et al. (1993) [50]	***	*	**	6
Yuan et al. (2008) [51]	***	*	**	6
Roncucci et al. (2008) [52]	****	*	**	7
Cui et al. (2009) [53]	***	**	*	6
Mclean al. (2011) [54]	****	*	**	7
Mariani et al. (2013) [55]	***	*	**	6
Jang et al. (2013) [56]	****	*	**	7
Hua et al. (2016) [57]	***	*	**	6
Maglietta et al. (2016) [58]	***	*	**	6
Zhu et al. (2016) [59]	***	*	*	5
Cui et al. (2017) [60]	***	*	**	6
Garcia et al. (2020) [61]	**	*	**	5
Chen et al. (2021) [62]	****	*	**	7
Omran et al. (2024) [63]	***	**	**	7
Cytokine- and other TiME-component-related studies				
Adegboyega et al. (2004) [64]	****	*	**	7
Cui et al. (2007) [65]	***	*	**	6
Cui et al. (2009) [66]	***	*	**	6
Cui et al. (2012) [67]	****	*	**	7
Wang et al. (2012) [68]	***	**	**	7
Cui et al. (2015) [69]	****	*	**	7
Xie et al. (2015) [70]	***	*	**	6
Cui et al. (2017) [71]	****	*	**	7
Cui et al. (2020) [72]	****	*	**	7
Cui et al. (2021) [73]	***	*	**	6
Youssef et al. (2021) [74]	****	*	**	7
Cui et al. (2022) [75]	***	*	**	6
Zhang et al. (2023) [76]	****	*	**	7
Relevant studies without a control group				
Moezzi et al. (2000) [77]	**	-	***	5
Kiziltaş et al. (2008) [78]	**	-	***	5
Freitas et al. (2021) [79]	**	-	***	5
Shams et al. (2021) [80]	**	-	***	5
Wallace et al. (2021) [81]	**	-	***	5
Wallace et al. (2021) [82]	**	-	***	5
Zhang et al. (2021) [83]	**	-	***	5

**Table 2:** A summary of human studies examining immune infiltration patterns in the TiME of conventional colorectal adenoma vs. normal mucosa or healthy controls and/or CRC tissue specimens along the A–C pathway.

Author (Publish Date)	NOS ≥5/9	Study Group Size (n)	Control Group Size (n)	Type of Cell Marker/Cytokine/Other Component	Detection Method	Clinical Evidence (Association with A, and/or CRC)
Human studies focused on tumor-infiltrating immune cell examination in conventional CRA vs. HC/NM (and CRC)						
Banner et al. (1993) [50]	6/9	HP: 16, A: tubular: 21, tubulovillous: 19, villous: 12, CRC: 17	NM: 27	UCHL-1+, L26+, IgG+, IgA+, S-100+, HLA-DR+, KP+, S-100+	IHC	The diffuse antigen-presenting system and activated immune response are shown in CRA and CRC vs. NM, represented by the expansion and reorganization of the T and B cell compartments and the TAM-cell system.
Yuan et al. (2008) [51]	6/9	A: 33 CRC: 23	HC: 19	mDCs (CD83+, CD208+); iDCs (CD1alpha+); COX-2, PGE2, receptors EP2/EP4	IHC, qRT-PCR, double IF, Colocalization Analysis	Altered DC infiltration along the ACS. Gradually increased COX-2 expression might contribute to the DC functional defect.
Roncucci et al. (2008) [52]	7/9	I: 65: A/CRC: 35, IBD: 8; II: 24 aberrant crypt foci: HP: 14, A: 16, CRC: 67	HC: 22	MPO+ cells	IHC	MPO+ cells (neutrophils and monocytes) gradually increase along the ACS, a potential marker of CRC risk. MSI seems to influence host immune responses to CRC.
Cui et al. (2009) [53]	6/9	A: 41, CRC: 25	HC: 15	Myofibroblasts, Lymphocytes, TAMs, COX-2	IHC, double IHC	Progressive cellular (lymphocytes, myofibroblasts, and COX-2) responses in the lamina propria could be involved in the ACS.
McLean et al. (2011) [54]	7/9	I: A: 65 II: A: LGD: 40, HGD: 40, Invasive CRC: 40	I: paired adjacent NM: 36; II: NM	CD3+, CD4+, CD8+, CD20+, CD25+, CD56+, CD68+; CXCL1, CXCL2, CXCL3, CCL20, IL8, CL23, CL19, CCL21, CCL5	IHC, RT-PCR	A phenotypic and genotypic ‘switch’ occurs early in the ACS, with the expression of inflammatory cytokines and chemokines dysregulated in the transition from NM → A, rather than from A → CRC.
Mariani et al. (2013) [55]	6/9	MA (LGD): 30 (11 patients), CRC 20 (60 samples)	HC: 20 (60 samples)	ThPOK+, CD4+, CD8+, CD56+, GZMB, RUNX3, FOXP3+	WBA, IF, qRT-PCR, Colocalization analysis	ThPOK may be considered a central regulator of the earliest events in the immune system during CRC development, decreasing the immune response against cancer cells.
Jang et al. (2013) [56]	7/9	HP: 15, A: LGD: 22, HGD: 27, Intramucosal CRC: 10, Invasive CRC: 32 (T2: 5; T3: 27)	Non-neoplastic mucosa: 17; Adjacent NM: 32	CD8+, FOXP3+, CD8+/Tregs ratio, COX-2, E-cadherin	IHC	Alteration in the distribution of both CD8+T cells (↓) and Tregs (↑) may generate an immune environment suitable for the development and progression of CRC.
Hua et al. (2016) [57]	6/9	A: 36 (♂: 26, ♀: 10; avg age: 65 yrs.); CRC: 30 (♂: 18, ♀: 12; avg age: 55.8 yrs.)	HC: 12 (♂: 7, ♀: 5)	FOXP3+, IL-10	IHC, RT-PCR	Expansion of regulatory T cells is an early event in ACS, presumably playing a role in regulating host immune response to the initiation of CRC.
Maglietta et al. (2016) [58]	6/9	I: 42: polypoid: 17, nonpolypoid: 25; II: 40: polypoid: 19, nonpolypoid: 21	I: 42 matched NM; II: None.	CD4+, CD8+, FOXP3+, MHC-I+, CD68+, CD163+	I: GSEA, II: IHC	The density of CD8+, FOXP3+, CD68+, CD163+, and MHC-I+ increases in the stroma of polypoid precancerous vs. nonpolypoid lesions. Large neoplasms have more immune cells in their stroma than small lesions. CD4+ increases along the conventional ACS in large polypoid vs. nonpolypoid lesions.
Zhu et al. (2016) [59]	5/9	A: 22, CRC: 48	NM: 21	CD4+, CD25+, FOXP3+, IL-10, Stat3	IHC	CD4^+^CD25^+^Foxp3^+^ Tregs inhibit tumor immunity in combination with IL-10 and Stat3. Expression of all three increases with CRC progression.
Cui et al. (2017) [60]	6/9	A: 30, CRC: 30	HC: 12	CD133+, LGR5+, ALDH1+, Musashi (Msi)+	IHC, double IHC	Changed temporal and spatial presentation of stem-like markers in different stages of human ACS.
Garcia et al. (2020) [61]	5/9	A: LGD: 58; HGD: 18	NM; FAP	CD3+, CD4+, CD8+, CD57+, CD68+, FOXP3.	IHC	Sporadic A contains a higher number of Treg cells, which suggests stronger immune selective pressure, while hereditary lesions have fewer immune infiltrates and seem to benefit from a more tolerant TiME.
Chen et al. (2021) [62]	7/9	Pre-cancer set: 62 (diverse sex, racial, and age groups): - Conventional A (tubular/tubulovillous), - Serrated polyps (SER) (HP/SSL); Cancer set: 93	NM: 66	CD4+, CD8+, CD68+, FOXP3+; Hypermutational status, WNT and serrated pathway activation genes	Multi-assay analysis: scRNA-seq, Whole Exome-seq, MxIF or MxIHC	Most immune cell types were increased in polyps vs. NM. Divergent tumor immune landscapes (in terms of immune cell densities, distribution in epithelial and stromal compartments, and spatial distribution in the glandular crypt and gene expression) in conventional vs. serrated CR lesions were established.
Omran et al. (2024) [63]	7/9	AA: 25, CRC: 25	HC: 19; adjacent NM	Expression of 579 immune genes genes (coding cells, cytokines, chemokines, receptors, etc.)	RT-qPCR	Early involvement in carcinogenesis (↑ TAMs, ↓ monocytes, ↑ activated mast cells, ↓ plasma B cells). A distinctive immunological signature in colorectal neoplasia, highlighting CXCL1, CXCL2, IL1B, IL6, CXCL8, PTGS2, and SPP1 as potential CRC biomarkers.
Human studies examining cytokine- and other TiME-component-related immune alterations in conventional CRA vs. HC/NM (and CRC)						
Adegboyega et al. (2004) [64]	7/9	HP: 43, sporadic A: 67, CRC: 39	NM: 50	SMA+, COX-2	IHC	Increased COX-2 expression (specifically to subepithelial intestinal myofibroblasts) is common in sporadic CRA, suggesting that myofibroblasts are important target cells for NSAID-mediated chemoprevention of CRC.
Cui et al. (2007) [65]	6/9	A: 32, CRC: 20	HC: 18	IL-4, IL-10, TNF-α, IFN-γ, IL-12A, IL-18	Q-PCR, IHC	Distinct changes in the Th1 cytokine profile (slightly increased in CRA and a remarkably decreased in CRC) may reflect a change in the host immune regulatory function along the ACS.
Cui et al. (2009) [66]	6/9	A: 53, CRC: 44	HC: 18	IL-8, receptors IL-8RA and IL-8RB	Q-PCR, IHC, double IHC	The activated IL-8 network in the TiME may function as a significant regulatory factor for the progression of A and AC transition.
Cui et al. (2012) [67]	7/9	A: 50, CRC: 50	HC: 15	IL-17A, Th17	qRT-PCR, s-q IHC	IL-17A and TH17 are highly activated throughout the CR ACS.
Wang et al. (2012) [68]	7/9	A: 31, CRC: 35	HC: 24; NM; tumor tissues ex vivo	IL-17A, Th17, anti-CD3, anti-CD28, IL-1β, IL-6, TGF-β, IL-21, IL-23	Flow cytometry, ELISA	A unique change of Th17 cells (↑), regulated by IL-1β, IL-6, and TGF-β in the progression of CRC.
Cui et al. (2015) [69]	7/9	A: 50; CRC: 50	HC: 30	IL-33, ST2	qRT-PCR, IHC	Elevated IL-33/ST2 axis expression along CR ACS might be involved in the neoplastic transformation via participation in the regulation of angiogenesis.
Xie et al. (2015) [70]	6/9	A: 8, CRC: 17, UC: 10	NM: 16	IL-17(R)A, ERK, VEGF(R), MMP9, MMP7, MMP2, Bcl-2, cyclin D1, BAX	ELISA, WBA, IHC	IL-17(↑) and its signaling pathways appear as promising new targets in the design and development of drugs for CRC prevention and treatment.
Cui et al. (2017) [71]	7/9	A: 50, CRC: 50	HC: 18	IL-21	qRT-PCR, Double IF	Increased IL-21 expression within the A/CRC TiME might have a potential predicting significance for survival time in patients with CRC.
Cui et al. (2020) [72]	7/9	A: 50, CRC: 50	HC: 30	IL-33, ST2, FOXP3+	qRT-PCR, IHC, Double IF	Increased densities of ST2-positive cells relate to Treg accumulation within the A/CRC TiME, suggesting the IL-33/ST2 pathway as a potential contributor to immunosuppressive milieu formation along the ACS.
Cui et al. (2021) [73]	6/9	A: 50, CRC: 45	HC: 15	IL-17A, Ki67, Myofibroblasts, CD146+	qRT-PCR, IHC, Double IF	The activated TH17/IL-17A network in the TiME is significantly associated with dynamic stromal cellular response throughout the ACS, which might provide a supportive environment for the initiation and progression of CRC.
Youssef et al. (2021) [74]	7/9	A: 29: LGD: 15, HGD: 14, CRC: 78	NM: 12	IL-8, TSP50, SERCA2	IHC	Increasing expression of IL-8, TSP50, and SERCA2 along ACS is associated with adverse prognostic factors through facilitating several hallmarks of cancer and could be considered independent prognostic factors.
Cui et al. (2022) [75]	6/9	A: 40, CRC: 37	HC: 21	IL-8, IL-1β, capacity of IL-1β to stimulate epithelial IL-8	q-PCR, IHC, double IF; ELISA	Activation of the IL-8 network in the niche of CSCs from the precancerous A stage to the CRC stage, which IL-1β potentially stimulates.
Zhang et al. (2023) [76]	7/9	HP: 30, A: LGD: 44, HGD: 29, CRC: 28.	HC: 29	CD4+, FOXP3+ TILs, and PD-1/PD-L1 immune checkpoints; ICOS, ICOSLG expression	IHC, multiple-IHC	Increased ICOS/ICOSLG expression may be associated with the progressive formation of FOXP3+TILs in the TiME and may further promote the development from precancerous neoplasia to CRC. Elevated co-expression of PD-1+ICOS+ or PD-1+ICOSLG+ contributes to the active TiME along the ACS.
Relevant human studies examining various immune infiltration patterns in CRA (and CRC along ACS), though lacking a control group						
Moezzi et al. (2000) [77]	5/9	HP: 65, A: 313, early CRC in A: 15, CRC: 95	None	TE%/all immune cells in the stroma	H&E, IHC	There is an inverse relationship between CRC’s invasiveness and stromal eosinophilia’s intensity.
Kiziltaş et al. (2008) [78]	5/9	HP: 96, SA: 50, A: 257, CRC: 45	None	TE%/all immune cells in the stroma	H&E, IHC	The intensity of TE is most prominent in A, including serrated adenomas, and is diminished along the ACS.
Freitas et al. (2021) [79]	5/9	-Sporadic: A: 60: LGD: 30, HGD: 30; CRC: 14; -FAP: 59: LGD: 30, HGD: 22; CRC: 7.	None	CD3+, CD4+, CD8+, FOXP3+, CD57+: TMB; MHC-I expression; PD-L1 expression	H&E, IHC, qPCR	The CR ACS is characterized by a progressive loss of adaptive immune infiltrate and by establishing a progressively immune cold microenvironment. It seems it is unrelated to the loss of tumor cells’ immunogenicity or the onset of an immunosuppressive TiME.
Shams et al. (2021) [80]	5/9	A: 22, CRC: 103	Non-neoplastic mucosa: 21	PD-L1+, CTLA-4+	16S	PD-L1+ and CTLA-4+ expression by tumor cells could cooperate in enhancing the progression of CRC, which could lead to poor patient prognosis. Conversely, their expression by TILs could stand against tumor progression.
Wallace et al. (2021) [81]	5/9	A: tubular: 21, tubulovillous: 37, villous: 36, serrated lesion: 7	None	CD117+, CD4+/RORC, MICA/B, IL6, IL17A, IFN-γ	IF	Proximal CR lesions were rich in immune infiltrates. The diminishing immune response with increasing villous histology suggests suppressive TiME.
Wallace et al. (2021) [82]	5/9	Caucasian Americans A (CaAs): 48, African Americans A (AaAs): 47	None	CD117+, CD4+/RORC, MICA/B, IL6, IL17A, IFN-γ	IF	Decreased immune responses in AaAs vs. CaAs may indicate impaired immune surveillance in early carcinogenesis. Proximal As are more common in AaAs.
Zhang et al. (2021) [83]	5/9	HP: 30, A: LGD: 44, HGD: 29, CRC: 50	None	Mutations of 10 genes: XRCC1, TP53, MLH1, MSH, KRAS, GSTP, UMP, THF, DPYD, ABCC2.	IHC, Transcriptome RNA	Increased IEL density and PD-1/PD-L1 expression correlate with cytological dysplasia progression, specifically with the XRCC1 mutation status in CRC, supporting a stepwise ACS and an XRCC1 hypermutated phenotypic mechanism of CR lesions.

NOS: Newcastle–Ottawa scale; HP: hyperplastic polyp; A: adenoma; AA: advanced adenoma; CR: colorectal; CRA: colorectal adenoma; CRC: colorectal cancer; LGD: low-grade dysplasia; HGD: high-grade dysplasia; ACS: adenoma–carcinoma sequence; TiME: tumor immune microenvironment; ♂: male; ♀: female; NM: normal mucosa; HC: healthy control; IBD: inflammatory bowel disease; MSI: microsatellite instable; MSS: microsatellite stable; ↑: increased/enhanced/activated; ↓: decreased/reduced/downregulated, →: indicates transition; avg: average; UCHL-1+: ubiquitin C-terminal hydrolase L-1+ T cells; L26+: B cells; S-100+: DCs (dendritic cells), HLA-DR+: T- cells; KP+: TAMs (tissue-associated macrophages), TMB: tumor mutation burden; MHC-I: major histocompatibility complex class I protein, showing nonspecific count of stromal cells; PD-L1: programmed death-ligand 1-immune checkpoint protein; CD3+: total T lymphocytes, CD4+: helper T lymphocytes, CD4+/RORC: Th17 cells; CD8+: cytotoxic T lymphocytes, FOXP3+: regulatory T cells (Tregs), CD57+: T lymphocytes/natural killer (NK) cells; CD56+: natural killer cells; CD25+: activated T cells; H&E: hematoxylin and eosin staining; IHC: immunohistochemistry; GSEA: gene set enrichment analysis; CD68+: macrophages M1, CD163+: macrophages M2; CD117+: mast cells; CD133+: multipotent stem cells; Msi (Musashi): stem-like cell marker; MICA/B: NK-cell ligands; MPO+ cells: neutrophils and monocytes; ThPOK: T helper-inducing POZ–Kruppel-like factor: a transcriptional regulator of T helper cell fate; GZMB: granzyme B; RUNX3: transcription factor; WBA: Western blot analysis; IF: immunofluorescence; qRT-PCR: quantitative real-time polymerase chain reaction; Q-PCR: quantitative real-time polymerase chain reaction; MA: microadenoma (dysplastic aberrant crypt foci); scRNA-seq: single-cell RNA sequencing; whole exome-seq: whole exome sequencing; s-q IHC: semi-quantitative immunohistochemistry; MxIF: multiplex immunofluorescence; MxIHC: multiplex immunohistochemistry; ELISA: enzyme-linked immunosorbent assay; SSL: sessile serrated lesion; mDCs (CD83+, CD208+): mature dendritic cells; iDCs (CD1alpha+): immature dendritic cells; PGE2: downstream signal molecule prostaglandin E2; COX-2: cyclooxygenase-2; ERK: extracellular signal-regulated kinase; TNF-α: tumor necrosis factor-alpha; IFN-γ: interferon-gamma; TSP50: Testes-specific protease 50 gene; MPO+: myeloperoxidase: neutrophils, monocytes; MMP: matrix metalloproteinase; Bcl-2: B cell lymphoma; BAX: Bcl-2-associated X protein; VEGF: vascular endothelial growth factor; CSCs: cancer stem-like cells, SMA: smooth muscle actin; IEL: intraepithelial lymphocytes, TE: tissue eosinophils, SA: serrated adenoma; FAP: familial adenomatous polyposis. Intramucosal carcinoma/carcinoma in adenoma/carcinoma in situ is defined as noninvasive CRC in clinical stage 0 according to AJCC. Early CRC is defined as CRC in clinical stage 1 and clinical stage 2 according to AJCC. Advanced CRC is defined as CRC in clinical stage 3 and clinical stage 4, according to AJCC. Advanced adenoma is defined as an adenomatous lesion that (a) has histologically proven high-grade dysplasia or/and (b) is ≥10 mm large or/and has a villous or tubulovillous component.

## Data Availability

Data available upon request.

## Supplementary Materials

The following supporting information can be downloaded at https://www.mdpi.com/article/10.3390/biomedicines13030699/s1, Table S1: PRISMA checklist, Table S2: PICOS, Table S3: Summary of included studies_extended version, Table S4: Detailed Newcastle–Ottawa Scale.
