# Supplementary material for: The Immune Environment in Colorectal Adenoma: A Systematic Review
Source: Biomedicines. 2025 Mar 12;13(3):699. doi: 10.3390/biomedicines13030699 (PMC11940254; doi:10.3390/biomedicines13030699)
Supplement: Supplementary file 1 [file biomedicines-13-00699-s001.zip › TableS4_Detailed Newcastle Ottawa Scale - corrected.pdf]

Table S4. Newcastle-Ottawa scale for quality assessment of immune cell-, cytokine- and other TiME components-related, and other relevant studies included in the systematic review. A study can be awarded a maximum of one star for each numbered item within the Selection and Exposure categories. A maximum of two stars can be given for Comparability.

	Case Definition (Histologically confirmed)	Representativeness of cases (n=100+)	Selection of controls (from the same area)	Definition of controls (Histological confirmation)	Comparability (Matched healthy controls and/or healthy part of the gut)	Exposure measurement (used internal control)	The same method for cases and controls	Non-response rate (Described or used consecutive recruitment)	Total score
Immune cell-related studies									
Banner et al. (1993) [50]	1	0	1	1	1	1	1	0	6
Yuan et al. (2008) [51]	1	0	1	1	1	1	1	0	6
Roncucci et al. (2008) [52]	1	1	1	1	1	1	1	0	7
Cui et al. (2009) [53]	1	0	1	1	1	1	1	0	6
McLean et al. (2011) [54]	1	1	1	1	1	1	1	0	7
Mariani et al. (2013) [55]	1	0	1	1	1	1	1	0	6
Jang et al. (2013) [56]	1	1	1	1	1	1	1	0	7
Hua et al. (2016) [57]	1	0	1	1	1	1	1	0	6
Maglietta et al. (2016) [58]	1	0	1	1	1	1	1	0	6
Zhu et al. (2016) [59]	1	0	1	1	1	0	1	0	5
Cui et al. (2017) [60]	1	0	1	1	1	1	1	0	6
Garcia et al. (2020) [61]	1	0	1	1	0	1	1	0	5
Chen et al. (2021) [62]	1	1	1	1	1	1	1	0	7
Omran et al. (2024) [63]	1	0	1	1	2	1	1	0	7
Cytokine- and other TiME components-related studies									
Adegboyega et al. (2004) [64]	1	1	1	1	1	1	1	0	7
Cui et al. (2007) [65]	1	0	1	1	1	1	1	0	6
Cui et al. (2009) [66]	1	0	1	1	1	1	1	0	6
Cui et al. (2012) [67]	1	1	1	1	1	1	1	0	7
Wang et al. (2012) [68]	1	0	1	1	2	1	1	0	7
Cui et al. (2015) [69]	1	1	1	1	1	1	1	0	7
Xie et al. (2015) [70]	1	0	1	1	1	1	1	0	6
Cui et al. (2017) [71]	1	1	1	1	1	1	1	0	7
Cui et al. (2020) [72]	1	1	1	1	1	1	1	0	7
Cui et al. (2021) [73]	1	0	1	1	1	1	1	0	6
Youssef et al. (2021) [74]	1	1	1	1	1	1	1	0	7
Cui et al. (2022) [75]	1	0	1	1	1	1	1	0	6
Zhang et al. (2023) [76]	1	1	1	1	1	1	1	0	7
Relevant studies without a control group*									

Moezzi et al. (2000) [77]	1	1	0	0	0	1	1	1	5
Kiziltaş et al. (2008) [78]	1	1	0	0	0	1	1	1	5
Freitas et al. (2021) [79]	1	1	0	0	0	1	1	1	5
Shams et al. (2021) [80]	1	1	0	0	0	1	1	1	5
Wallace et al. (2021) [81]	1	1	0	0	0	1	1	1	5
Wallace et al. (2021) [82]	1	1	0	0	0	1	1	1	5
Zhang et al. (2021) [83]	1	1	0	0	0	1	1	1	5

* These studies should be carefully evaluated due to a higher risk of bias.
