# Supplementary material for: The Immune Environment in Colorectal Adenoma: A Systematic Review
Source: Biomedicines. 2025 Mar 12;13(3):699. doi: 10.3390/biomedicines13030699 (PMC11940254; doi:10.3390/biomedicines13030699)
Supplement: Supplementary file 1 [file biomedicines-13-00699-s001.zip › TableS3_Summary of included studies_extended version - corrected.pdf]

**Table S3**. Summary of human studies examining cell-, cytokine- and other component-related immune changes in the microenvironment of conventional colorectal adenoma vs. normal mucosa or healthy control (or without a control group*) and/or colorectal cancer tissue specimens.

| **Author (publish date)** | **NOS ≥5/9** | **Study group size (n)** | **Control group size (n)** | **Type of cell marker/cytokine/other component** | **Detection method** | **Main Findings// Clinical evidence** (association with A, and/or CRC) |
|---|---|---|---|---|---|---|
| Human studies examining tumor-infiltrating immune cells in conventional CRA microenvironment vs. HC/NM (and CRC along ACS) | | | | | | |
| **Banner et al. (1993) [50]** | 6/9 | HP: 16,<br>A: tubular: 21,<br>tubulovillous: 19, villous: 12,<br>CRC: 17 | NM: 27 | UCHL-1+, L26+, IgG+, IgA+, S-100+, HLA-DR+, KP+, S-100+ | IHC | UCHL-1-+ T-cells: > in the lamina propria of all specimens.<br>In A and CRC: reactive lymphoid follicles composed of L26+ B-cells, UCHL-1+ T-cells, and ↑UCHL-1-+ T-cells in the epithelial compartment.<br>KP1+ TAMs, S-100+ DCs, and HLA-DR+ cells oriented toward the lumen in NM and HP.<br>↑TAMs, DCs, and HLA-DR expression in ACS in neoplasms (> in the stroma). |
| **Yuan et al. (2008) [51]** | 6/9 | A: 33<br>CRC: 23 | HC:19 | mDCs (CD83+, CD208+);<br>iDCs (CD1alpha+);<br>COX-2, PGE2, receptors EP2/EP4 | IHC,<br>qRT-PCR,<br>double IF,<br>Colocalization Analysis | mDCs: ↓ A→ ↓ CRC vs. HC; iDCs: ↑ A→ ↑↑ CRC vs. HC.<br>A: mDCs abundantly distributed in the subepithelial stroma of A;<br>CRC: the distribution of mDCs in the tumor stroma was not homogeneous, more frequently found in invading edges; ↑iDCs in the intratumoral mass, and some infiltrated into the malignant epithelium.<br>COX-2 expression: ↑ A→ ↑↑ CRC vs. HC.<br>Colocalization of PGE2 receptors EP2/EP4 with mDCs in the stroma of CRC. |
| **Roncucci et al. (2008) [52]** | 7/9 | I: 65: A/CRC: 35,<br>IBD: 8;<br>II: 24 aberrant crypt foci: HP: 14, A: 16, CRC: 67 | HC: 22 | MPO+ cells | IHC | MPO+(neutrophils-monocytes) cell number:↑A:↑LGD→↑↑HGD→↑↑↑ CRC (MSI > MSS) vs. HC. |
| **Cui et al. (2009) [53]** | 6/9 | A: 41,<br>CRC: 25 | HC: 15 | Myofibroblasts, Lymphocytes, COX-2 | IHC,<br>double IHC | Proliferation labeling index in lamina propria cells: ↑A→↑↑CRC vs. HC;<br>↑Lymphocytic infiltration in both the lamina propria and dysplastic (A) epithelium;<br>↑Myofibroblasts in the lamina propria.<br>Intraepithelial TAMs: ↑A→ ↑↑CRC vs. HC;<br>COX-2 expression: ↑A→ ↑↑CRC in the tumor lamina propria vs. HC. |
| **McLean et al. (2011) [54]** | 7/9 | I: A: 65<br>II: LGD: 40; HGD: 40;<br>CRC: 40 | I: paired adjacent NM: 36;<br>II: NM | CD3+, CD4+, CD8+, CD20+, CD25+, CD56+, CD68+; CXCL1, CXCL2, CXCL3, CCL20, IL8, CCL23, CCL19, CCL21, CCL5 | IHC,<br>RT-PCR | TAMs, neutrophil, T helper, activated T and NK cells: ↑ in A vs. adjacent NM;<br>LGD→HGD→invasive CRC:↑CD68+;<br>Infiltration of TAMs, neutrophils, and activated T cells correlated with A size.<br>Expression of CXCL1, CXCL2, CXCL3, CCL20, and IL-8: ↑A and CRC vs. NM;<br>Expression of CCL19, CCL21, CCL23, CCL5: ↓A and CRC vs. NM. |
| **Mariani et al. (2013) [55]** | 6/9 | 60 samples from 20 CRC patients;<br>30 MA (LGD) from 11 patients (1 sample FFFE, other 2 fresh frozen -80*C/per patient); | HC: 20 (60 samples) | ThPOK+, CD4+, CD8+, CD56+, GZMB, RUNX3, FOXP3+ | WBA,<br>IF,<br>qRT-PCR,<br>Colocalization Analysis | CD4+T helper lymphocytes: NM →~MA→↓CRC;<br>CD8+ T cytotoxic lymphocytes: NM → ↓MA→↓↓CRC;<br>CD56+ NK cells: NM →↑MA→↑↑CRC;<br>ThPOK+: NM→ ↑MA→↑↑CRC. |
| **Jang et al. (2013) [56]** | 6/9 | HP: 15, A: LGD: 22, HGD: 27, Intramucosal CRC: 10, Invasive CRC: 32 (T2: 5; T3: 27) | Non-neoplastic muco sa: 17; | CD8+, FOXP3+, CD8+/Tregs ratio, COX-2, E-cadherin | IHC | Tregs: ↑A (HGD)→↑↑CRC vs. non-neoplastic mucosa; |

| | | | | | | |
|---|---|---|---|---|---|---|
| | | | Adjacent NM: 32 | | | CD8+ T cells and CD8+ T cells/Tregs ratio: ↓A (HGD)→↓↓ CRC vs. non-neoplastic mucosa; ↓in the tumor center of A (LGD and HGD); in invasive CRC associated with gender, differentiation, node metastasis, and tumor budding;<br>COX-2 expression: ↑A (HGD)→↑↑CRC and positively associated with Tregs infiltration. |
| **Hua et al. (2016) [57]** | 6/9 | A: 36 (♂: 26, ♀: 10; average age: 65 yrs.);<br>CRC: 30 (♂: 18, ♀: 12; average age: 55.8 yrs.) | HC: 12<br>(♂: 7, ♀: 5) | FOXP3+, IL-10 | IHC,<br>RT-PCR | FOXP3+cells: NM→ ↑↑ A→↑↑↑CRC;<br>FOXP3+: in lamina propria of NM>> in epithelium; in stroma of A>>in epithelium; in tumor stroma of CRC>>in epithelium;<br>IL-10 in A and CRC was expressed in tumor stromal and epithelial cells.<br>IL-10: ↑↑ A vs. HC; ↑CRC vs. HC. |
| **Maglietta et al. (2016) [58]** | 6/9 | I: 42 lesions: polypoid: 17 (9 small, 8 large), and nonpolypoid: 25 (10 small, 15 large);<br>II: 40 lesions: polypoid: 19 (10 small, 9 large), nonpolypoid: 21 (10 small, 11 large).<br>. | I: 42 matched samples of NM;<br>II: None. | CD4+, CD8+, FOXP3+, MHC-I+, CD68+, CD163+ | I: GSEA,<br>II: IHC | CD4+: ↑A→↑↑ CRC vs. normal mucosa; ↑ with size and grade of dysplasia;<br>↑↑CD4+ cells in large polypoid tumors vs. nonpolypoid counterparts;<br>FOXP3+ (Treg) and CD8+ T cell densities did not increase significantly with lesion size (esp. in polypoid lesions);<br>CD8+, FOXP3+, CD68+, CD163+, and MHC-I+: ↑polypoid vs. nonpolypoid lesions vs. NM (independently of lesion size);<br>↑CD4+, MHC-I+, and CD68+ in large nonpolypoid lesions;<br>CD8+/CD4+ ratios: 0.4 (small polypoid), 0.3 (large polypoid), and 0.23 (nonpolypoid lesions of all sizes). |
| **Zhu et al. (2016) [59]** | 5/9 | A: 22, CRC: 48 | NM: 21 | CD4+, CD25+, FOXP3+, IL-10, Stat3 | IHC | FOXP3+(Tregs), IL-10 and Stat3: ↑A→ ↑↑↑CRC vs. NM.<br>FOXP3+ positively correlated with Stat3 at the mRNA level, with IL-10 at the protein level.<br>FOXP3+ in CD4$^{+}$CD25$^{+}$FOXP3$^{+}$Tregs correlated with the histological grade, lymph node metastasis, and TNM stage of CRC; IL-10 with the histological grade and TNM stage, Stat3 with the lymph node metastasis and TNM stage. |
| **Cui et al. (2017) [60]** | 6/9 | A: 30,<br>CRC: 30 | HC: 12 | CD133+, LGR5+, ALDH1+, Musashi (Msi) | IHC,<br>double IHC | Stem-like markers positive cells: ↑↑↑ A→ ↑↑↑CRC and expanded to the middle part of the transitional crypt in A and CRC.<br>LGR5+ and ALDH1+ cells:↑↑↑ in A/CRC epithelium and are associated with the degree of dysplasia in the A, and node involvement in the CRC.<br>Proliferative stromal cells labeled by PCNA (vimentin+ fibroblasts): ↑A and CRC stroma. |
| **Garcia et al. (2020) [61]** | 5/9 | A: LGD: 58; HGD: 18 | NM;<br>FAP | CD3+, CD4+, CD8+, CD57+, CD68+, FOXP3+. | IHC | FOXP3+ and CD68+: ↑ LGD-sporadic A vs. NM and vs. FAP;<br>FOXP3+ and CD4+: ↑↑HGD-sporadic A vs. FAP. |
| **Chen et al. (2021) [62]** | 7/9 | Pre-cancer sample set:<br>- discovery set: 65 specimens, 30 tumors;<br>- validation set: 63 specimens, 32 tumors.<br>62 tumors from diverse sex, racial, and age groups:<br>-Conventional A (tubular/ tubulovillous), | NM: 66 | CD4+, CD8+, CD68+, FOXP3+, Hypermutational status, WNT and serrated pathway activation genes | Multi-assay analysis: scRNA-seq, Whole Exome-seq,<br>MxIF or MxIHC | Most immune cell types were ↑ in polyps vs. NM.<br>CD8+, NK cells, and labeled cytotoxic γδTcells, CD8+/ CD4+: ↑↑ in SER vs. A.<br>FOXP3+ activity was ↑ in A-derived vs. NM CD4+ T cells.<br>CD8+ T cells infiltrated into the epithelial compartments of SER.<br>CD68+ cells were distributed throughout the stroma in conventional A, concentrating at the luminal surfaces in SER. |

| | | | | | | |
|---|---|---|---|---|---|---|
| | | -Serrated polyps (SER) (HP/ SSL);<br>Cancer sample set: 93 | | | | |
| **Omran et al. (2024) [63]** | 7/9 | Norwegian cohort:<br>AA: 25, CRC: 25 | HC: 19;<br>adjacent NM | Expression of 579 immune genes (coding cells, cytokines, chemokines, receptors, etc.) | RT-qPCR | TAMs: ↑AA→ ↑↑CRC; Monocytes: ↓AA→↓↓CRC;<br>Mast cells become activated in AA and CRC;<br>↓plasma B-cells (↓IgG): ↑memory B-cells in AA, ↓naïve B-cells in AA and CRC.<br>Expression of CXCL1, CXCL2, IL-1B, IL-6, CXCL8 (IL8), PTGS2, and SPP1: ↑CRC vs. HC; expression of CXCL1, CXCL2, IL-6, CXCL8, and PTGS2: ↑AA vs. HC. |
| Human studies examining cytokine- and other TiME components-related immune alterations in conventional CRA vs. HC/NM (and CRC along ACS) | | | | | | |
| **Adegboyega et al. (2004) [64]** | 7/9 | HP: 43, A: 67,<br>CRC: 39 | NM: 50 | SMA+,<br>COX-2 | IHC | COX-2 in A: localized to SMA+ (myofibroblasts) in the periluminal region of the lamina propria vs. NM (+/- HP): intact epithelium, COX-2 only in TAMs and endothelial cells.<br>In CRC: myofibroblasts were limited to the adenomatous portion of the tumor, detected in 62% of cases. COX-2 by malignant epithelial cells was observed in 23% of CRC. |
| **Cui et al. (2007) [65]** | 6/9 | A: 32,<br>CRC: 20 | HC: 18 | IL-4, IL-10, TNF-α, IFN-γ, IL-12A, IL-18 | Q-PCR,<br>IHC | IFN- γ, TNF-α, IL-12A, and IL-18: ↑A→ ↓↓CRC vs. HC.<br>IL-4, IL-10: ↑A→ ↑↑CRC.<br>Cytokine-expressing cells: polarized to the subepithelial stroma in A, evenly distributed through the stroma in CRC. |
| **Cui et al. (2009) [66]** | 6/9 | A: 53,<br>CRC: 44 | HC: 18 | IL-8,<br>receptors IL-8RA and IL-8RB | Q-PCR,<br>IHC,<br>double IHC | IL-8 mRNA level: ↑A→ ↑↑ CRC vs. HC;<br>IL-8 and its receptors were observed both in the stroma and in the A/CRC cells.<br>IL-8 expression was characterized in macrophages, lymphocytes, and myofibroblasts in the tumor stroma. Co-expression of IL-8RA and IL-8RB with CD34+ tumor-associated micro-vessels in both the A and CRC. |
| **Cui et al. (2012) [67]** | 7/9 | A: 50,<br>CRC: 50 | HC: 15 | IL-17A,<br>Th17 | qRT-PCR,<br>s-q IHC | IL-17A: ↑A→ ↑↑CRC (in both stroma and epithelium) vs. HC;<br>↑IL-17A is associated with the severity of dysplasia and expression of TH17-stimulating factors throughout the ACS. |
| **Wang et al. (2012) [68]** | 7/9 | A: 31,<br>CRC: 35 | HC: 24;<br>NM;<br>tumor tissues *ex vivo* | IL-17A, Th17, Anti-CD3, anti-CD28, IL-1β, IL-6, TGF-β, IL-21, IL-23. | Flow cytometry,<br>ELISA | Th17 cells: ↑A (circulation); ↑CRC (tissues); Treg cells: ↑ CRC (circulation).<br>IL-1β, IL-17A, IL-23: ↑A→↑↑CRC;<br>IL-6: ↓CRC; TGF-β, IL-17A: ↑CRC. |
| **Cui et al. (2015) [69]** | 7/9 | A: 50;<br>CRC: 50 | HC: 30 | IL-33,<br>ST2 | qRT-PCR,<br>IHC | IL-33/ST2 mRNA: ↑↑↑A→↑↑CRC vs. HC (in both the tumor stromal cells and A/CRC cells).<br>Expression level of ST2 in CRC was associated with tumor/node/metastasis (TNM) stage; ↑IL-33+ and ST2+ micro-vessels were found in the stroma of A and CRC. |
| **Xie et al. (2015) [70]** | 6/9 | A: 8,<br>CRC: 17,<br>UC: 10 | NM: 16 | IL-17(R)A,<br>ERK, VEGF(R), MMP9, MMP7, MMP2, Bcl-2, cyclin D1, BAX | ELISA,<br>WBA,<br>IHC | IL-17A: A→↑CRC; ↑UC vs. NM;<br>Receptor (IL-17RA): ↑↑CRC > ↑A & UC vs. NM;<br>IL-17A and IL-17RA were accompanied by ↑ERK and c-Jun N-terminal kinase (JNK) pathways, ↑in the expression of MMP9, MMP7, MMP2, Bcl-2, and cyclin D1, ↓in BAX expression, and ↑ in VEGF and VEGF(R). |
| **Cui et al. (2017) [71]** | 7/9 | A: 50,<br>CRC: 50 | HC: 18 | IL-21 | qRT-PCR,<br>Double IF | IL-21: ↑A→↑↑CRC (stroma) vs. HC;<br>IL-21 level was correlated with the overall survival time in CRC patients;<br>IL-21+ cells were mostly NK cells and T lymphocytes in the tumor stroma. |
| **Cui et al. (2020) [72]** | 7/9 | A: 50,<br>CRC: 50 | HC: 30 | IL-33, ST2,<br>FOXP3+ | qRT-PCR,<br>IHC,<br>Double IF | ST2 and FOXP3+ mRNAs: ↑A→↑CRC vs. HC.<br>ST2+ cell densities: ↑A→↑CRC (epithelium and stroma), associated with ↑FoxP3+ Tregs densities; |

| | | | | | | |
|---|---|---|---|---|---|---|
| | | | | | | ST2+ cell density in the tumor stroma was associated with a degree of dysplasia in patients with A, and disease stages and lymph node metastasis in patients with CRC. Kaplan-Meier survival curves: CRC with ↑ST2+cells in the stroma have shorter overall survival. |
| **Cui et al. (2021) [73]** | 6/9 | A: 50, CRC: 45 | HC: 15 | IL-17A, Ki67, Myofibroblasts, CD146+ | qRT-PCR, IHC, Double IF | IL-17A: ↑A→↑↑ CRC. ↑Ki67 and ↑myofibroblasts in the A/ CRC stroma; ↑IL-17 receptor C, one of the key elements for the IL-17 receptor complex, was highly expressed in CD146+ A/CRC stromal cells. |
| **Youssef et al. (2021) [74]** | 7/9 | A: 29 (LGD: 15, HGD: 14) CRC:78 | NM: 12 | IL-8, TSP50, SERCA2 | IHC | IL-8, TSP50, SERCA2: ↑A→↑↑↑CRC vs. NM., associated with the degree of dysplasia in A; with the lympho-vascular invasion, advanced TNM staging, and high intra-tumoral inflammatory infiltrate in CRC, overall, with worse prognosis. |
| **Cui et al. (2022) [75]** | 6/9 | A: 40, CRC: 37 | HC: 21 | IL-8, IL-1β, capacity of IL-1β to stimulate epithelial IL-8 | q-PCR, IHC, double IF; ELISA | Expression of CSCs and IL-8, IL-1β transcript: ↑A and CRC epithelium vs. HC. Co-expression of the IL-8 receptors (IL-8RA and IL-8RB) with LGR5 labeled CSCs in CRC tissue sections. |
| **Zhang et al. (2023) [76]** | 7/9 | HP: 30, A: LGD: 44, HGD: 29, CRC: 28 | HC: 29 | CD4+, FOXP3+ TILs, and PD-1/PD-L1 immune checkpoints; ICOS and ICOSLG expression | IHC, multiple-IHC | ICOS/ICOSLG, CD4+/Foxp3+ TILs, PD-1/PD-L1: ↑A→↑↑CRC. Location and expression level of ICOS/ICOSLG may be involved in precancerous lesion-carcinoma progression. PD-1 together with ICOS/ ICOSLG expression status stratifies patients with CR lesions into low, moderate, and high risk for progression. ↑PD-1+ICOS+/PD-1+ICOSLG+ strongly correlates with CRC. |
| Relevant human studies examining various immune infiltration patterns in CRA (and/or CRC along ACS), though lacking control group | | | | | | |
| **Moezzi et al.*(2000) [77]** | 5/9 | HP: 65, A: tubular: 176, tubulovillous: 55, villous: 82, early CRC in adenoma: 15, CRC: 95 | None | TE%/all immune cells in the stroma | H&E, IHC | TE: ↑CRC transitional zone (between normal tissue and carcinoma)→↓↓↓CRC stroma. Stromal TE: A↑↑→CRC↓. |
| **Kiziltaş et al.* (2008) [78]** | 5/9 | HP: 96, SA; 50, A: Flat: 19, tubular: 154, tubulovillous: 71, villous: 13, CRC: 45 | None | TE%/all immune cells in the stroma | H&E, IHC | TE: ↑A(LGD=HGD)→CRC↓↓; TE: ↓HP; ↑↑SA. |
| **Freitas et al.*(2021) [79]** | 5/9 | Sporadic A: 60: LGD: 30; HGD: 30, CRC: 14; FAP: 59: LGD: 30; HGD: 22, CRC: 7 | None | CD3+, CD4+, CD8+, FOXP3+, CD57+: TMB, MHC-I & PD-L1 expression | H&E, IHC, qPCR | CD3+, CD4+, CD8+, FOXP3+, CD57+: ↓A→ ↓↓CRC (sporadic & FAP); FOXP3+: sporadic-LGD > FAP-LGD; PD-L1 expression: ↓CRC stroma (sporadic & FAP); PD-L1 expression: ↓earlier FAP vs. sporadic lesions; TMB & MHC-I expression: ↑ sporadic lesions. |
| **Shams et al.* (2021) [80]** | 5/9 | A: 22, CRC: 103 | Non-neoplastic mucosa (from intussusception, volvulus, diverticulitis): 21 | PD-L1+, CTLA-4+, | 16S | PD-L1+ epithelial expression: ↑A (HGD) & CRC vs. non-neoplastic mucosa; CTLA-4+ epithelial expression: ↑A (HGD & LGD) & CRC vs. non-neoplastic mucosa. ↑PD-L1+TILs and CTLA-4+TILs: association with absence of perforation, early T stage, pushing TBC, lower TB score, high TSR, and presence of peritumoral lymphocytes. Prolonged survival: associated with ↓PD-L1+ and CTLA-4+ epithelial expression, ↑PD-L1+TILs and ↑CTLA-4+TILs. |

| | | | | | | |
|---|---|---|---|---|---|---|
| **Wallace et al.*(2021) [81]** | 5/9 | A: tubular: 21, tubulovillous: 37, villous: 36, serrated lesion: 7 | None | CD117+, CD4+/RORC, MICA/B, IL-6, IL-17A, IFN- γ | IF | CD117+, CD4+/RORC, MICA/B, IL-6, IFN-γ: ↑↑↑in proximal colon A vs. distal colon and rectum A;<br>CD117+, MICA/B, IL-6, IL-17A, IFN-γ: ↓↓↓ villous histology; LGD=HGD. |
| **Wallace et al.*(2021) [82]** | 5/9 | Caucasian Americans A (CaA): 48, African Americans A (AaA): 47 | None | CD117+, CD4+/RORC, MICA/B, IL-6, IL17A, IFN- γ | IF | Proximal A: AaAs > CAs; Rectal A: AaAs< CaAs;<br>IFN-γ, NK cell ligand: ↓AaAs vs. CaAs (in age, sex, and batch-adjusted models);<br>CD4, NK cell ligands, Th17, mast cells, and IFN-γ: ↓AaAs vs. CaAs (in models adjusted for age, sex, and clinicopathologic variables). |
| **Zhang et al.*(2021) [83]** | 5/9 | HP: 30,<br>A: LGD: 44, HGD: 29,<br>CRC: 50 | None | Mutations of 10 genes: XRCC1, TP53, MLH1, MSH, KRAS, GSTP, UMP, MTHF, DPYD, ABCC2. | IHC, Transcriptome RNA seq | CD4+FOXP3+: ↑A→↑↑CRC;<br>↑PD-L1 expression and PD-1 expression within the IELs.<br>↑XRCC1 expression within tumor nuclei;<br>CR lesions: left side >> right side. |

NOS: Newcastle-Ottawa scale; HP: hyperplastic polyp; A: adenoma; AA: advanced adenoma; CR: colorectal; CRA: colorectal adenoma; CRC: colorectal cancer; LGD: low-grade dysplasia; HGD: high-grade dysplasia; ACS: adenoma-carcinoma sequence; TiME: tumor immune microenvironment; ♂: male; ♀: female; NM: normal mucosa; HC: healthy control; IBD: inflammatory bowel disease; MSI: microsatellite instable; MSS: microsatellite stable; >: more/prevalent/dominate; < less/rare; ↑: increased/enhanced/activated; ↓: decreased/reduced/downregulated, →: indicates transition; avg: average; UCHL-1+: ubiquitin C-terminal hydrolase L-1+ T cells; L26+: B-cells; S-100+: DCs (dendritic cells), HLA-DR+: T- cells; KP+: TAMs (tissue-associated macrophages), TMB: tumor mutation burden; MHC-I: major histocompatibility complex class I protein, showing nonspecific count of stromal cells; PD-L1: programmed death-ligand 1-immune checkpoint protein; CD3+: total T lymphocytes, CD4+: helper T lymphocytes, CD4+/RORC: Th17 cells; CD8+: cytotoxic T lymphocytes, FOXP3+: regulatory T cells (Tregs), CD57+: T lymphocytes/ natural killer (NK) cells; CD56+: natural killer cells; CD25+: activated T cells; H&E: hematoxylin and eosin staining; IHC: immunohistochemistry; GSEA: gene set enrichment analysis; CD68+: macrophages M1, CD163+: macrophages M2; CD117+: mast cells; CD133+: multipotent stem cells; Msi (Musashi): stem-like cell marker; MICA/B: NK-cell ligands; ThPOK: T helper-inducing POZ–Kruppel-like factor: a transcriptional regulator of T helper cell fate; GZMB: granzyme B; RUNX3: transcription factor; WBA: Western Blot Analysis; IF: immunofluorescence; qRT-PCR: quantitative real time-polymerase chain reaction; Q-PCR: quantitative real-time polymerase chain reaction; FFPE: Formalin-Fixed Paraffin-Embedded; MA: microadenoma (dysplastic aberrant crypt foci); scRNA-seq: single-cell RNA sequencing; whole exome-seq: whole exome sequencing; s-q IHC: semi-quantitative immunohistochemistry; MxIF: multiplex immunofluorescence; MxIHC: multiplex immunohistochemistry; ELISA: enzyme-linked immunosorbent assay; SSL: sessile serrated lesion; NSAIDs: Non-steroidal anti-inflammatory drugs; mDCs (CD83+, CD208+): mature dendritic cells; iDCs (CD1alpha+): immature dendritic cells; PGE2: downstream signal molecule prostaglandin E2; COX-2: cyclooxygenase-2; ERK: extracellular signal-regulated kinase; PBMC: peripheral blood mononuclear cells; TNF-α: tumor necrosis factor-alpha; IFN-γ: interferon-gamma; TSP50: Testes-specific protease 50 gene; MMP: matrix metalloproteinase; Bcl-2: B-cell lymphoma; BAX: Bcl-2-associated X protein; VEGF: vascular endothelial growth factor; CSCs: cancer stem-like cells, SMA: smooth muscle actin; IEL: intraepithelial lymphocytes, TE: tissue eosinophils, SA: serrated adenoma; FAP: Familial Adenomatous Polyposis.

Intramucosal carcinoma/Carcinoma in adenoma/carcinoma in situ is defined as noninvasive CRC in clinical stage 0 according to AJCC. Early CRC is defined as CRC in clinical stage 1 and clinical stage 2,i according to AJCC. Advanced CRC is defined as CRC in clinical stage 3 and clinical stage 4 according to AJCC. Advanced adenoma is defined as an adenomatous lesion that: a) has histologically proven high-grade dysplasia or/and b) is ≥10 mm large or/and has a villous or tubulovillous component.
