# Supplementary material for: The Immune Environment in Colorectal Adenoma: A Systematic Review
Source: Biomedicines. 2025 Mar 12;13(3):699. doi: 10.3390/biomedicines13030699 (PMC11940254; doi:10.3390/biomedicines13030699)
Supplement: Supplementary file 1 [file biomedicines-13-00699-s001.zip › TableS2_PICOS.pdf]

Table S2. *PICOS* of the included studies in the systematic review.

**Parameters**	**Inclusion criteria**
Population	Adult patients with pathologically confirmed conventional CRA (+/- CRC) and being tested for dysplastic lesion-associated colorectal tissue immune infiltration.
Intervention	Mucosal tissue sampling for the evaluation of immune landscapes in polypoid premalignant colorectal lesions.
Comparison	(a) Control group:<br>(1) gut tissue of a healthy population and/or;<br>(2) same patient's healthy adjacent mucosa;<br>(b) CRC group (assessment along the adenoma-carcinoma sequence)
Outcomes	(1) Immune cell-related infiltration in conventional CRA microenvironment versus healthy control/normal mucosa (+/-CRC);<br>(2) Cytokine- and other immunological factor-related immune alterations in conventional CRA versus healthy control/normal mucosa (+/- CRC) tissues;<br>(3) Other studies*;<br>(4) Significant linkage between immune changes in sporadic precancerous colorectal lesions and polyp-associated characteristics (morphology, size, grade of dysplasia, and location in the gut)
Study design	Nonrandomized clinical trials, retrospective comparative, and prospective observational studies.
Date	From inception to December 2024
Language	English

CRA: colorectal adenoma
CRC: colorectal cancer
TiME: tumor immune microenvironment
* Relevant studies, though without an appropriate control group
